# Application of Artificial Intelligence-Based Regression Methods in the Problem of COVID-19 Spread Prediction: A Systematic Review

**DOI:** 10.3390/ijerph18084287

**Published:** 2021-04-18

**Authors:** Jelena Musulin, Sandi Baressi Šegota, Daniel Štifanić, Ivan Lorencin, Nikola Anđelić, Tijana Šušteršič, Anđela Blagojević, Nenad Filipović, Tomislav Ćabov, Elitza Markova-Car

**Affiliations:** 1 Faculty of Engineering, University of Rijeka, Vukovarska 58, 51000 Rijeka, Croatia; jmusulin@riteh.hr (J.M.); dstifanic@riteh.hr (D.Š.); ilorencin@riteh.hr (I.L.); nandelic@riteh.hr (N.A.); 2 Faculty of Engineering, University of Kragujevac, Sestre Janjić, 34000 Kragujevac, Serbia; tijanas@kg.ac.rs (T.Š.); andjela.blagojevic@kg.ac.rs (A.B.); fica@kg.ac.rs (N.F.); 3 Bioengineering Research and Development Centre (BioIRC), Prvoslava Stojanovića 6, 34000 Kragujevac, Serbia; 4 Faculty of Dental Medicine, University of Rijeka, Krešimirova ul. 40, 51000 Rijeka, Croatia; tomislav.cabov@fdmri.uniri.hr; 5 Department of Biotechnology, University of Rijeka, Radmile Matejčić 2, 51000 Rijeka, Croatia; elitza@biotech.uniri.hr

**Keywords:** AI-based methods, COVID-19, open-access data, spread modeling

## Abstract

COVID-19 is one of the greatest challenges humanity has faced recently, forcing a change in the daily lives of billions of people worldwide. Therefore, many efforts have been made by researchers across the globe in the attempt of determining the models of COVID-19 spread. The objectives of this review are to analyze some of the open-access datasets mostly used in research in the field of COVID-19 regression modeling as well as present current literature based on Artificial Intelligence (AI) methods for regression tasks, like disease spread. Moreover, we discuss the applicability of Machine Learning (ML) and Evolutionary Computing (EC) methods that have focused on regressing epidemiology curves of COVID-19, and provide an overview of the usefulness of existing models in specific areas. An electronic literature search of the various databases was conducted to develop a comprehensive review of the latest AI-based approaches for modeling the spread of COVID-19. Finally, a conclusion is drawn from the observation of reviewed papers that AI-based algorithms have a clear application in COVID-19 epidemiological spread modeling and may be a crucial tool in the combat against coming pandemics.

## 1. Introduction

Coronavirus disease 2019 (COVID-19) is a viral infection that has held the attention of the worldwide public for over a year and will certainly be remembered as one of the crucial events which had shaped the decade before us. It is caused by a member of the Betacoronavirus family—Severe Acute Respiratory Syndrome Virus 2, known as SARS-CoV-2 [1,2]. Most of the current research shows that the spread of the disease began in the Wuhan province, China in late 2019 [3]. As with many infective diseases of its nature, efforts have been made to model the spread of the disease and predict the epidemiological curves [4]. Different applications have been considered—either as forewarning systems of future spread velocity [5], development of tools to enable the officials to determine strategies in combating the spread of COVID-19 [6], or prediction of COVID-19’s influence in different areas such as economics [7], education [8], or transport [9]. Some researchers have applied numerous classical modeling methods in an attempt to determine the spreading models of COVID-19. Some of this research has included Tsallis and Tirnakli proposing q-statistical functional form [10], Vespignani et al. proposing the use of exploitation of natural variation in the distribution of the cases, dynamic mathematical modeling, basic reproduction number R0, and others [11], while Ziff shows the application of fractal kinetics [12]. Seeman et al. applied a genomics approach [13], with Thurner et al. opting for a network-based modeling approach [14]. Still, due to various influencing parameters which may be hard to include and determine, or may be overlooked by the researchers, many of the models show shortcomings [15]. Therefore, a different approach may be necessary.

AI-based regression models may provide the required capabilities. Regression is a method, commonly used in many disciplines, for the creation of models which can predict the value of an output variable based on the set of input variables [16,17]. For complex regression models, AI-based methods are often used. This type of regression method enables the detection of complex relations between input variables, as well as the automatic detection of interactions between input variables and the output [18]. Such an approach has shown to be successful in the modeling of previous epidemics, including but not limited to SARS [19], H1N1 [20,21], avian flu [22,23], AIDS [24,25], and Ebola [26,27]. Thus, it is evident that the AI-based approach may greatly help with the modeling of COVID-19 epidemiological curves. This review paper will provide an overview of the application of AI-based methods in the COVID-19 spread prediction.

Many existing reviews of data science applications in fields related to COVID-19 have been published. Vaishya et al. [28] focus the review on general applications of AI in combat versus COVID-19. Naude [29] provides one of the earliest reviews of AI application in multiple areas: early warnings and alerts, tracking and prediction, data dashboards, diagnosis and prognosis, treatments and cures, and social control. Tayarani and Mohhamad [30] provide a later review, which also focuses on various fields—including not only epidemiology, but also diagnosis and treatment applications of AI as well. Agbehadji et al. [31] focus their review on the papers which apply data science analytics and AI in the field of detection and contact tracing. Adly et al. [32] focus on the review of the applicability of existing research in the field of the Internet of Things in combination with AI techniques. Ahmad et al. [33] focus on the review of papers that utilize ML in the prediction of the number of infected patients.

The presented review differentiates from the existing ones in multiple key points. First is the focus on papers that apply AI in the field of COVID-19 spread, without limiting the review on a specific goal (such as the number of infections or deaths), allowing for a wider review. Second is the inclusion of EC algorithms as one of the groups of algorithms selected for the review which are often overlooked in previous reviews. Finally, the presented review includes the number of preprints, which include early manuscripts, and papers accepted for publication but are not yet published. This was done due to the importance of rapid modeling in such applications of COVID-19, where early findings may be crucial because of the developing and changing nature of pandemics. Offering a systematic review of the papers which focus on the mentioned points is the main motivation of this review paper, with the additional motive being the provision of an overview of the available datasets, the researchers can use for epidemiological modeling of COVID-19. Available data are a key component of any data-driven AI-based approach in research, and public COVID-19 datasets have not been given large importance in existing reviews. The main questions that this paper tries to address may be summed as:

- Which publicly available datasets can be used for AI-based research in the field of COVID-19 spread prediction?
- Are there applications of EC algorithms for COVID-19 spread prediction, and how do they compare to ML algorithms result-wise?
- Which are the most commonly used algorithms and evaluation techniques in the COVID-19 modeling?
- What are the results of preprints in the AI-based regression modeling of COVID-19 spread, and how do they compare to the published work?

The manuscript is organized in five sections. First, the methodology is presented—including the field taxonomy and PRISMA. This is followed by two sections containing overviews of the researched data, first describing the publicly available datasets, and second describing the reviewed research items. For readability, the review of research items is split into ML and EC sections, with further subcategories being used where appropriate. Finally, the authors provide observations on noticed trends and give conclusions based on the performed systematic review.

## 2. Methods

As mentioned in the introduction, this systematic review was performed by using the PRISMA 2020 statement [34] as a guideline. The detailed specification of methods used during the process of systematic reviewing is presented in Table 1.

**Figure 1:**
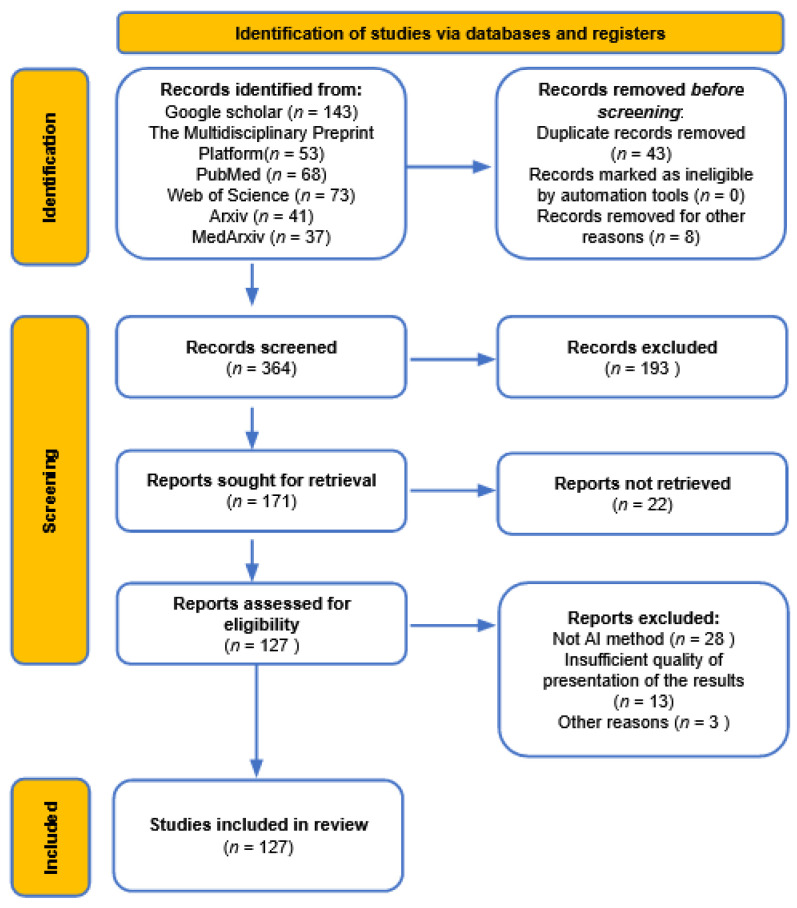
Visualization of the reviewing procedure using PRISMA flowchart.

To visualize the reviewing procedure, the PRISMA flowchart is provided in Figure 1, where each number represents the number of studies used in different syntheses.

**Figure 2:**
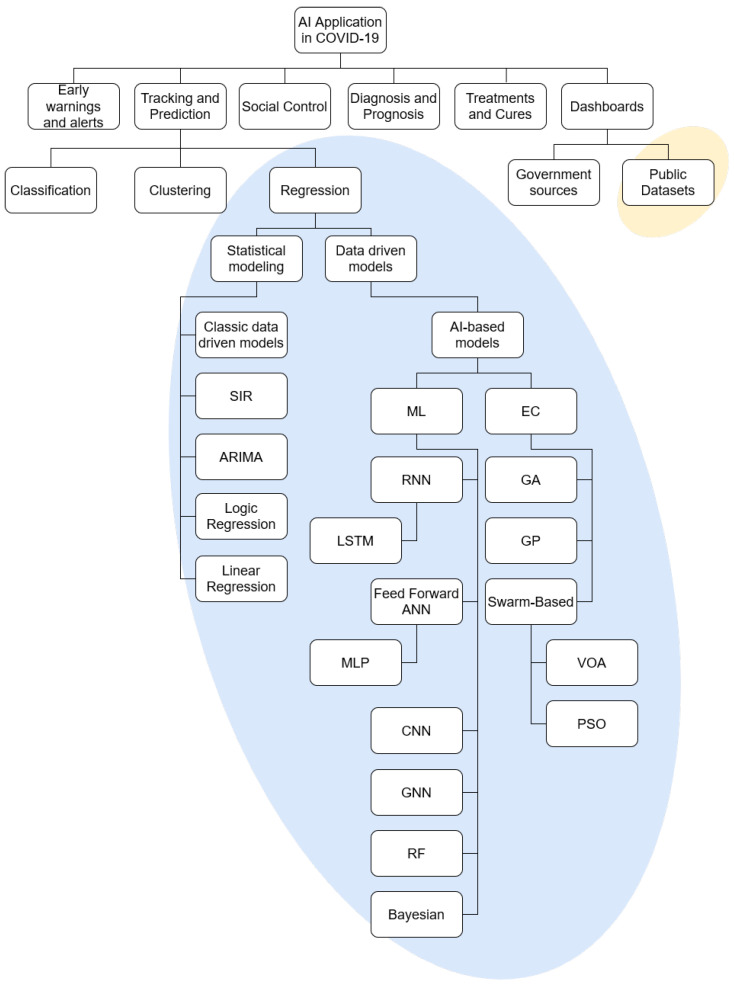
Taxonomy of the AI research in the field of COVID-19, with the research reviewed in this paper being marked with an ellipsis. SIR-Suspectible, Infectious or Recovered, ARIMA-Autoregressive Integrated Moving Average, RNN-Recurrent Neural Network, LSTM-Long Short-Term Memory, ANN-Artificial Neural Network, MLP-Multilayer Perceptron, CNN-Convolutional Neural Network, GNN-Graph Neural Network, RF-Random Forest, GA-Genetic Algorithm, GP-Genetic Programming, VOA-Virus Optimization Algorithm, PSO-Particle Swarm Optimization.

The field of AI application in COVID-19 is vast and it may be split into multiple subfields [29]. To make the focus of the paper more apparent, and illustrate the topics used in the review, the taxonomy is given in Figure 2. The taxonomy of AI application in COVID-19 is based on previous research in the field [29,31]. The split amongst the methods within the regression field is done according to the established taxonomy of such methods [35,36,37]. The field in which the review has been performed has been marked with the elliptical background elements.

## 3. COVID-19 Datasets

A key part of the application of AI-based regression techniques is data that can be used to fit the models trained by the algorithms. The data need to be plentiful and represent the real situation as well as possible, considering that any errors in the data may cause errors in the predictions of models. This section presents some of the most commonly used datasets for the epidemiological spread of COVID-19. The datasets in question are collected from various local government agencies. Each of the presented datasets lists the sources used. Some of the common sources, for the countries with a high number of cases, are:

- Center for Disease Control and Prevention in USA (CDC) [38],
- Robert Koch Institute in Germany [39],
- Protezione Civile and Ministero della Salute in Italy [40],
- Instituto de Salud Carlos III in Spain [41],
- National Health Commission of the People’s Republic of China (NHC) [42], and
- Brazil Ministry of Health [43].

World Health Organization (WHO) is an independent specialized agency of the United Nations, whose task is to help achieve the highest level of health for all people in the world; it is headquartered in Geneva [44]. WHO is responsible for managing global health issues, setting standards, designing health research and development programs, monitoring and assessing health trends, providing technical support to countries, and defining strategic documents based on scientific evidence. On 11 March 2020, the WHO declared COVID-19 as a global pandemic. On the WHO official website is a dashboard with the number of global confirmed cases and deaths, collected daily [45]. These data are official and have high precision. The data may be downloaded in a table format, with data sorted by country in alphabetical order and each country data sorted daily since 3 January 2020. The is separated into columns consisting of:

- date of the report DATE,
- country code (CC),
- country,
- WHO region the country belongs to ($WHO_{R}$),
- number of new cases since the last daily report ($C_{N}$),
- number of cumulative cases since the start of reporting ($C_{C}$),
- number of new deaths since the last daily report ($D_{N}$), and
- the number of cumulative deaths since the start of reporting ($D_{C}$).

**Figure 3:**
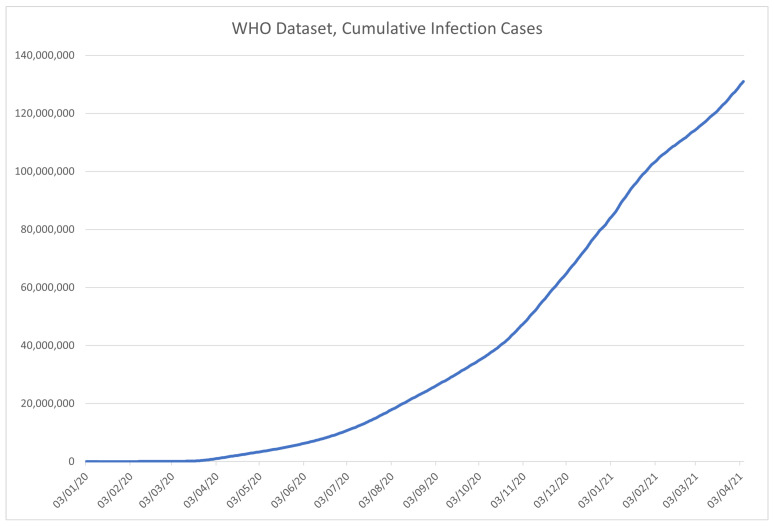
Time-series plot of the data in the WHO dataset, for the number of COVID-19 infections, contained within the dataset.

**Figure 4:**
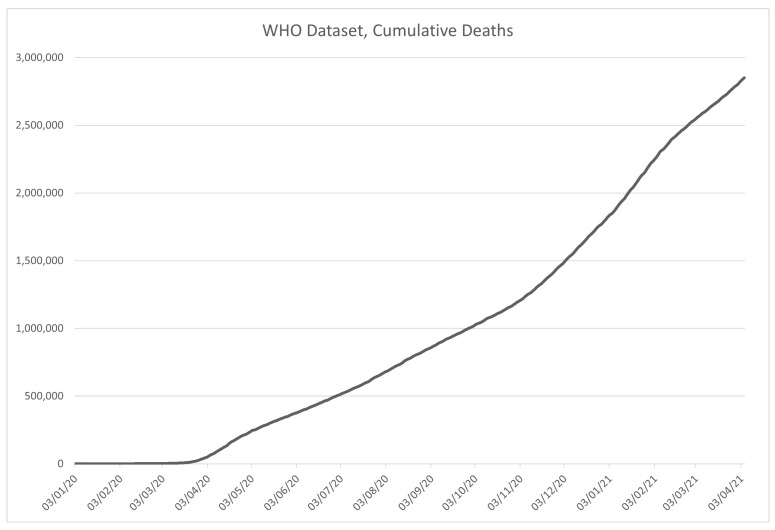
Time-series plot of the data in the WHO dataset, for the number patient deaths caused by COVID-19, contained within the dataset.

An excerpt from the data in the WHO dataset is given in Table 2. The columns are given in order described in the previously given list and marked with corresponding codes, with the visualization of the data in the dataset shown in Figure 3 for the number of confirmed COVID-19 cases, and Figure 4 for the deceased patient data.

John Hopkins University (JHU) is a private research university founded in 1876 in Baltimore, Maryland [46]. An interactive map created by the Center for Systems Science and Engineering at the renowned University of Maryland shows exactly how many confirmed cases of COVID-19, deaths, and recovered patients are in the world. JHU presented its interactive map for the first time on 22 January 2020 [47,48]. To create such a detailed overview, JHU scientists collect data from the WHO, regional and state ministries of health, and local media reports. The website is designed to provide researchers, government institutions, and the public, a tool to monitor the spread of infection in real-time. The data displayed are made available publicly inside a GitHub repository and are updated daily. The data are available in [49] and are still regularly updated. Data is formatted in three time-series tables, for the number of confirmed, recovered, and deceased patients per day per country. An excerpt from the JHU dataset is given in Table 3, with “…” representing skipped dates, not shown in the presented data example.

**Figure 5:**
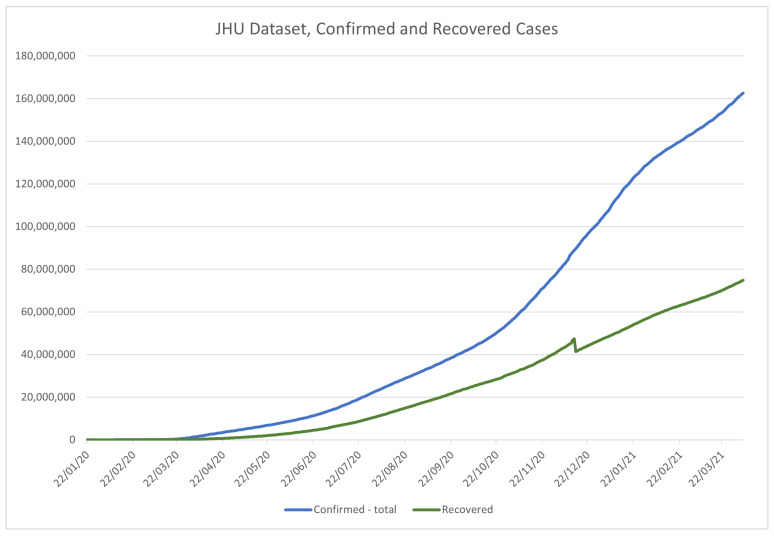
Time-series plot of the data in the JHU COVID-19 dataset, for confirmed and recovered patients, contained within the dataset.

**Figure 6:**
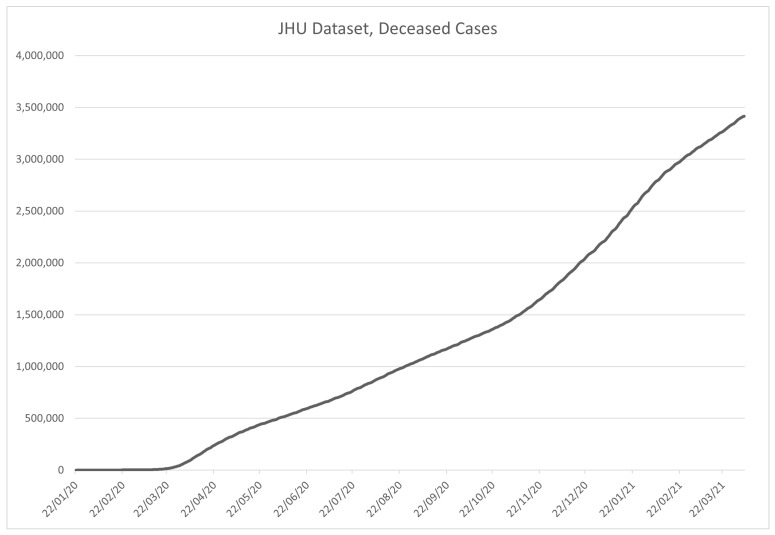
Time-series plot of the data in the JHU COVID-19 dataset, for deceased patients, contained within the dataset.

JHU dataset is popular among the researchers for many reasons, including the convenient time-based formatting for each country, regular updates, and precision. As a result of the large amount of data, the dataset has been split into global and US datasets, allowing for more precise, per-county data collection for the US. Figure 5 and Figure 6 show the data from the global dataset of the JHU, in the period from 22 January 2020 to 17 February 2021, for recovered ($N_{R}$) and confirmed ($N_{C}$), and deceased cases ($N_{D}$), respectively. The value of the above is that the number of active cases ($N_{A}$) can be derived from the above data using:

(1) $$N_{A}=N_{C}-\left(N_{R}+N_{D}\right)$$

**Figure 7:**
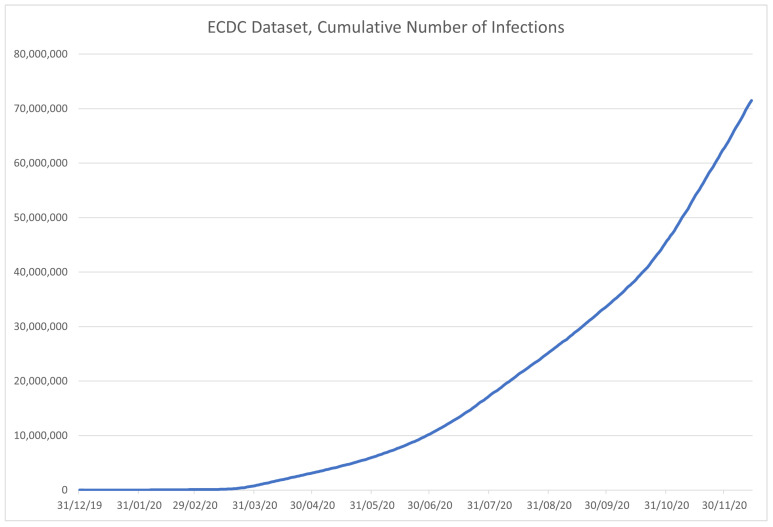
Time-series plot of the data in the ECDC dataset, for confirmed patients, contained within the dataset.

**Figure 8:**
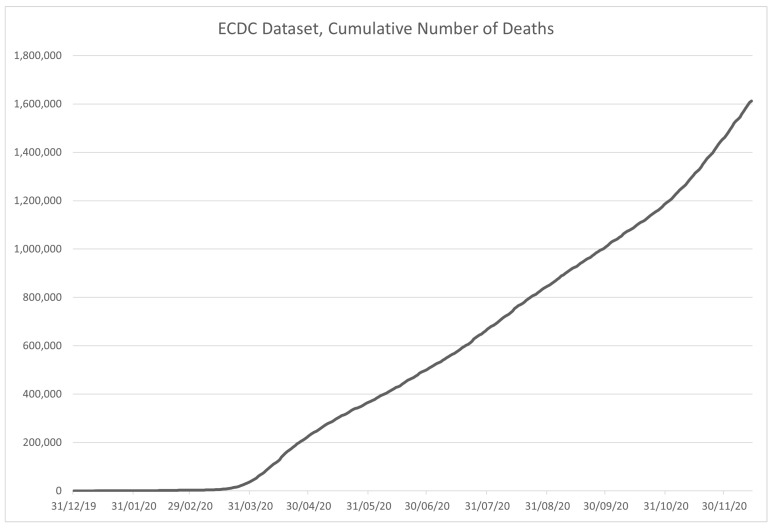
Time-series plot of the data in the ECDC dataset, for deceased patients, contained within the dataset.

The role of the European Centre for Disease Prevention and Control (ECDC) is to strengthen the European defense against communicable diseases [50]. It provides scientific advice to EU governments and institutions, ensures early detection and analysis of upcoming threats to the EU, it helps EU member state governments prepare for disease outbreaks, analyzes and interprets data obtained from the EU Member States on 52 communicable diseases and conditions. The dataset is available at [51], but it is no longer updated since ECDC has switched to weekly instead of daily reporting since 14 December 2020. The data are sorted by country (column “Countries and territories”-Country), and contain the date—in both formatted and separated formats, along with the number of newly reported cases (*C*) and deaths (*D*) for the given date. Along with that information, the dataset contains geoID of the country, country territory code (CC), population data for the country collected in 2019 (POP), a continent the country is on, and the cumulative number of COVID-19 cases per 100,000 people in population for 14 days. The example of the data contained in the dataset is given in Table 4, with the data contained in the dataset being shown in Figure 7 for the infected, and in Figure 8 for the deceased patients.

Worldometer website [52] provides detailed data on the number of cases per country, with excellent tracking of the number of active cases, recovered cases, deaths, and other metrics per country. Data are provided in a tabular format for the daily updates, containing the numbers of new cases and cumulative cases, while the historical data are displayed as graphs; with data being sourced from WHO. Still, the data are not made easily available for download in a tabular format, which makes the use of it harder for researchers. This dataset has been used in some initial research [53,54], but as time goes on the complexity of data collection from the website increases, making previously mentioned datasets an easier resource to utilize.

Many research items exist in the field of serological prevalence of COVID-19 in patients [55,56,57]. Some of this research indicates that the numbers of patients are much higher in reality, than suggested by data contained in the public datasets [58]. Public datasets of serological prevalence are also available, such as from CDC [59] and Our World in Data [60]. Not many researchers have utilized this data for AI-based spread modeling, possibly due to lower publicity of such sets in comparison to datasets that were given an overview in this paper.

## 4. Modeling of COVID-19 Using SIR and ML Methods

In this section, an overview of ML-based methods for COVID-19 spread modeling will be provided. ML is a field of AI which uses data to adjust and train models which are then tested on previously unseen data to determine their efficiency. While capable of providing high-quality models, with extremely high regression quality and low errors, ML models suffer from the fact that a lot of data points are needed to train them. As it can be seen from the previous section, a large amount of epidemiology-related data is generated by the COVID-19 pandemic—enabling the use of ML methods.

### 4.1. SIR and Similar Methods

Since the start of the COVID-19 pandemic, much research has been devoted to predicting the outbreak by utilizing different mathematical modeling approaches, including, but not limited to, classical susceptible-infective-recovered (SIR) model and its derivatives, susceptible-exposed-infective-recovered (SEIR), and other general-purpose models. The classical SIR model is based on three Ordinary Differential Equations (ODE), by which, the susceptible, infective, and recovered populations can be expressed.

Muñoz-Fernández et al. [61] used a classical SIR-type model with non-constant parameters to construct a mathematical model which predicts the evolution of COVID-19. The model is tested on official data from Italy, Spain, and the USA. Model’s prediction for Italy and the USA were quite negative, which means that daily new cases of deaths and confirmed cases will grow. Sedaghat et al. [62] use the susceptible-infectious-recovered-deceased (SIRD) model to predict trends of COVID-19 in Kuwait. As the model input, COVID-19 data for 20, 40, 60, 80, 100, and 116 days are used. To obtain optimal parameters of the SIRD model, a MATLAB Isqcurvefit optimization algorithm is utilized. According to the presented results, the peak infectious day can be predicted after 40 days. However, in terms of sensitivity analysis, the SIRD model is not very accurate. In conclusion, a SIRD model can be used for rough estimations of COVID-19 peak infectious day. Ivorra et al. [63] developed a new mathematical model, θ-SEIHRD, for the spread of COVID-19 in China. θ-SEIHRD model considers special characteristics of the disease, such as the existence of undetected cases of infected people as well as various infectious and sanitary conditions of people in the hospital. The novelty of their approach stands in the ratio of fraction θ of detected cases over a total number of real infected cases. The developed model resulted in a 4.2732 reproduction ratio (results obtained with experiment EXP29M), which is higher than values reported in other relevant studies. Furthermore, Ivorra et al. [64] developed a θ-SEIQHRD model to simulate dynamics of COVID-19 and possible future scenarios in Italy. Authors included time-series dynamic coefficients, undetected deaths, which mostly occur in nursing homes, effects of different control measures as well as quarantine and people in hospital. Results indicate that there will be new outbreaks if the control measures are too much relaxed. Badr et al. [65] based on epidemiological data, compute the growth rate ratio of COVID-19 for a given US county on a particular day. In their study, authors used mobility ratio for each day and county to quantify how social distancing affected the rate of new infections. The analysis is focused on 25 counties, and the data do not include sociodemographic information. Obtained results indicate that social distancing has been crucial in reducing the growth rate in several counties in the United States. To forecast the COVID-19 epidemic in India and high incidence states, Malavika, B. et al. (2021) [66] used a logistic growth curve and SIR models. The data for India were obtained from the “covid19india.org” while the data for other countries were obtained from Kaggle. First, the logistic growth model was utilized for short-term prediction; second, SIR models were used to forecast the maximum number of active cases and peak time; third, the impact of lockdown on the incidence of new COVID-19 cases was evaluated utilizing a Time Interrupted Regression (TIR) model. According to the presented results, the logistic growth curve model achieved accurate predictions in terms of a short-term scenario for India and high incidence states (Maharashtra, Tamil Nadu, Delhi, and Rajasthan). The authors stated that prediction obtained by the SIR model can be considered as a warning signal for preparing the health systems. Moreover, the results imply that immediately after the lockdown, there is no significant decrease in the number of COVID-19 daily cases. Singh and Gupta (2021) [67] extended the classical SIR model and proposed a Generalized SIR (GSIR) model to monitor the COVID-19 pandemic. Such a model is an integrative model, by which multiple waves of COVID-19 daily cases can be encompassed. The study was performed utilizing the proposed model on the COVID-19 data for Brazil, India, the USA, and the World. Obtained results showed that the GSIR model is better performing compared to the results of the classical SIR model. According to the authors’ conclusion, continuous predictive monitoring of COVID-19 can be achieved utilizing the proposed GSIR model.

However, Moein et al. [68] in their research show inefficiency of SIR models in forecasting the COVID-19 pandemic in Isfahan. According to the presented results, the SIR model was unable to accurately forecast the COVID-19 pandemic in the long term. Part of the reason why the SIR model fails in forecasting the actual spread lies in the simplicity of the model itself, by which, important features and factors that directly or indirectly affect the course of the disease are ignored. According to the proposed methodology in [63,64], developed models are only suitable for places with a relevant number of infected people. Additionally, some limitations that occur are in human behaviors, which are not predictable as cells or molecules. Moreover, the effect of the temperature and humidity on COVID-19 spreading has not been considered, as well as poor-quality data due to undocumented infection cases. More limitations were stated in [65] where some mitigating factors, such as handwashing and wearing face masks, the difference between low-risk and high-risk trips, and limited testing capacity are not accounted for analysis. To forecast the COVID-19 pandemic effectively, a large amount of precise data are required along with more advanced mathematical approaches that consider various factors which affect the course of the disease.

### 4.2. Use of Feed-Forward Neural Networks

One of the earliest published works in the application of epidemiological curve modeling using AI is by Car et al. [69]. The epidemiological curves have been modeled globally, using data from many locations. In the paper, the researchers have applied a Multilayer Perceptron (MLP) Artificial Neural Network (ANN) regressor. The dataset, which was obtained as a time series dataset, has been transformed into a regression dataset using the number of days elapsed since the start of the infection, as well as the longitude and latitude of each geographic location in the dataset. Three separate models have been trained by the authors—separate models for the number of confirmed, recovered, and deceased cases. The results have been cross-validated using a K-fold algorithm (5 folds have been used in the research) and evaluated using the coefficient of determination ($R^{2}$). Authors have achieved $R^{2}$ scores of 0.94 (σ = 0.037) for the model of the confirmed cases, 0.986 (σ = 0.021) for the model of the deceased cases, and 0.781 (σ = 0.072) for the model of the recovered cases. Sujath et al. [70] applied multiple techniques to the prediction of COVID-19 spread inside India. Data utilized in the study are collected from publicly available repositories on Kaggle, with Weka and Orange frameworks utilized for data preprocessing and filtering. The authors utilize the MLP, linear regression, and vector autoregression on the collected data. Authors conclude that the data collected can be used with the above methods in the prediction of the numbers of confirmed, deceased, and recovered cases in the short-term periods following the data collection. The conclusion drawn is that to achieve precise predictions, the data collection and modeling process should be performed continuously. Chakraborty and Ghosh [71] aimed to solve two goals in their presented research: short-term real-time forecasting of the number of confirmed cases of COVID-19 and risk assessment in terms of the death rate. Modeling for both goals has been performed for various countries. The first goal, the forecasting model, is developed using a hybrid approach using an ARIMA model and a wavelet-based forecasting (WBF) model. The 10-day forecasts have been developed for Canada, France, India, South Korea, and the UK. The novel proposed hybrid model overcomes the issues faced with singular models generated by both methods. The hybrid model has been set up as a pipeline in which the ARIMA model generates the residual series which are then used in the WBF. This approach greatly improves the forecasting of models when evaluated using Mean Absolute Error (MAE) and Root Mean Square Error (RMSE). Risk assessment is performed for 50 cases exposed to a high risk from COVID-19 using a Regression Tree (RT) model. The method in question is used to detect the influence of individual inputs. The results achieved by the method are RMSE of 0.013 and $R^{2}$ of 0.896. Chen [72] demonstrated the training of predictive models for Taiwan. The author compares multiple methods: Threshold Autoregressive Models (TAR), Smooth Transition Autoregressive Models (STAR), and ANN models. The author concludes that the NN achieves a narrower confidence interval in comparison to the TAR and STAR derived models, with the results also being graphically evaluated in comparison to the real data. The author concluded that the growth of the infected cases in Taiwan is, at the time, stationary and should not increase exponentially soon. Mollalo et al. [73] developed the models for coronavirus incidence rates across the continental area of the United States of America, using ANNs. The model of incidence rates allows for the prediction of coronavirus case instances through time. The authors collected a database of 57 potential explanatory variables and applied the MLP ANN. The authors conclude that even a simple MLP, with a single hidden layer, could explain 65% of the correlation between the input variables and the predicted incidence rates. The authors use the developed models for a sensitivity analysis which allowed them to conclude that age-adjusted mortality rates of ischemic heart disease, leukemia, and pancreatic cancer, median household income, and total precipitation are the input variables with the highest influence. The authors also applied the logistic regression and determined that the presence/absence of the incidence hotspots are explainable with the aforementioned variables at a statistically significant rate. This was concluded using Getis-Ord Gi* test with *p* < 0.05.

Kumar et al. [74] have forecasted the COVID-19 pandemic dynamics with ARIMA and ML. The authors utilize the methods for the data obtained by 30 April 2020 in 15 countries selected for the highest number of confirmed COVID-19 cases globally. At the time of the research, these countries were: United States, China, Italy, Spain, Germany, Iran, France, Switzerland, United Kingdom, South Korea, Netherlands, Austria, Belgium, Canada, and Turkey. Authors apply pre-processing techniques such as data smoothing to remove short-term changes in time series. Authors predict the quick rise of the confirmed cases in the upcoming months and suggest that stringent measures be implemented to curb the spread of COVID-19 disease. Allah and Hassanien [75] proposed the ISACL-MFNN forecasting model, trained on the data since 22 January 2020. The proposed model is based on the Chaotic Learning (CL) Interior Search Algorithm (ISA), being integrated into a multi-layer feed-forward ANN (MFNN). The ISA is enhanced using CL, allowing for the avoidance of local optima and this combination is used to adjust the hyperparameters of the MFNN. The data utilized for the training are obtained from WHO and the research focuses on the prediction of confirmed cases. Authors evaluate the solution using MAE, RMSE, MAPE, root mean square relative error (RMSRE), and $R^{2}$. with the model being applied in the countries with high infection rates—USA, Italy, and Spain. The results of the proposed model show an increase in the prediction quality in comparison to unoptimized MFNN and MFNN optimized with different algorithms. The research presented by Hasan [76] has used a hybrid ANN model based on ensemble empirical mode decomposition (EEMD) to model COVID-19 spread using data collected between 22 January and 18 May 2020. The regression performances were evaluated by using Mean square error (MSE) and $R^{2}$ metrics. By using the presented methodology, $R^{2}$ scores up to 0.99982 and MSE scores as low as $3.76\times 10^{-5}$ were achieved on the testing dataset. This research has confirmed a possibility for ANN utilization in COVID-19 spread modeling. A local approach was also proposed by Saba et al. [77], where ML algorithms were utilized to forecast the prevalence of COVID-19 outbreaks in Egipt. The aforementioned approach was enabled by using autoregressive ANNs trained with data collected between 1 March and 10 May 2020. By using these methods, high-performance estimations were obtained achieving values of 7.752, 10.410, and 0.999 for MAE, RMSE, and $R^{2}$ scores, respectively. Presented results showed the possibility of utilization of similar algorithms in forecasting COVID-19 spread in local environments. Pontoh et al. [78] have proposed an ANN-based approach to estimate the effectiveness of the public health measures in Jakarta and West Java. To define the method with the best forecasting performances, multiple ANN methods were designed, and these are MLP, ANN Auto-Regressive, and Extreme Learning Machine. The results have shown that an approach based on MLP, which consists of two hidden layers with 10 neurons, achieved the highest forecasting performances. When the aforementioned configuration is used for estimation of COVID-19 spread in Jakarta and West Java, it can be concluded that restrictive measures have provided the reduction of COVID-19 spread. Vaid et al. [79] have proposed a method for the estimation of unobserved cases of COVID-19 infections. The method consists of dimensionality reduction and an unbiased hierarchical Bayesian estimator. The presented method has shown that the number of unobserved cases largely exceeds the number of confirmed cases (1.6 million vs. 840,476 for the USA and 60,000–86,000 vs. 41,650 for Canada). Alakus and Turkoglu [80] have performed a comparison of multiple ANN-based algorithms for the prediction of COVID-19 occurrence by using laboratory data. These algorithms were designed by combining CNN, RNN, and LSTM. The aforementioned algorithms have achieved an accuracy of 86.66%, F1-score of 91.89%, the precision of 86.75%, recall of 99.42%, and AUC of 62.50%. The authors have concluded that proposed algorithms could be employed to assist medical experts in the validation of laboratory findings.

Melin et al. [81] present the multiple ensemble ANN model with fuzzy response aggregation for predicting the COVID-19 time series in Mexico. With the ensemble ANNs, the predictions under different conditions can be produced, and by utilizing fuzzy logic, the responses of these neural predictors can be aggregated. The dataset was obtained from Mexico’s Government website and consists of confirmed and death cases for 12 states in Mexico, along with confirmed and death cases for the entire country. Experimental results of the multiple ensemble ANN models with fuzzy response integration show significantly better values of performance measures than those obtained using traditional monolithic ANNs. To predict the peak of COVID-19 in Spain, Baltas et al. [82] propose an AI method based on deep ANNs. Firstly, Monte Carlo simulations of SIR epidemiology models are used for the data generation process. After the data generation, a prediction model based on deep ANNs is designed. Input data for the period from 9 March 2020, to 25 March 2020, were used to train the DNN model since in that period the rapid growth of infected people was observed. As a model performance measure, MAE and MAPE were used. Experimental results show that the estimated peak of infected people is 79 days after the first COVID-19 confirmed case in Spain. Farooq et al. [83] propose an ANN-based adaptive online incremental learning technique to build a model of the COVID-19 pandemic. In such an approach, a model is intelligently adapted whenever new input data is received. India was taken as a research object on which the model was validated to demonstrate the effectiveness of the proposed method. By utilizing the developed model, the authors also investigate the impact of preventive measures on the evolution of COVID-19 disease. As a result, an effective method is proposed by which the number of death cases caused by the pandemic can be reduced. Pereira et al. [84] demonstrate the use of Deep Learning tools to predict the dynamics of transmission of COVID-19 by analyzing contamination data. Data are publicly available and collected from JHU. As the main contribution authors demonstrate a way to train a modified auto-encoder (MAE) to forecast COVID-19 spreading. Auto-encoder is a particular type of ANN architecture that is trained to copy its input to its output. Furthermore, results show that the pandemic is still growing in Brazil and the predicted number reaches a total of 240 thousand infected Brazilians. Another AI-based approach for estimation of COVID-19 spread was proposed by Ndiaye [85]. In this case, an AI-based approach was utilized to predict the volume of COVID-19 spread worldwide and in China, Italy, Iran, and Senegal, with the lowest achieved result, evaluated with MRE, being given in the paper at 4.20% for China. Pinter et al. [86] apply ANFIS and MLP-ICA methods on the problem of predicting the number of infected individuals and mortality rates of COVID-19 outbreak. Validation is performed on the period of 9 days, with models achieving $R^{2}$ scores of 0.99 when MLP-ICA algorithm is used.

The overview of selected papers is given in Table 5. Papers for which the results were evaluated numerically, instead of just visually, are repeated for comparison of results achieved through the use of feed-forward neural networks, such as Multilayer Perceptron.

### 4.3. Use of Recurrent Neural Networks

Tomar and Gupta [88] demonstrated the utilization of curve fitting methods, and ANNs such as Long Short-Term Memory (LSTM) models to achieve a 30-day prediction of various parameters, such as the total number of confirmed cases, total recovered cases, total number of deceased cases and the daily number of positive cases. Authors used data from 30 January to 4 April 2020 to train the models, with 80% of the data used for training and 20% used for testing. Authors also modeled the influence of various measures taken to combat the spread of COVID-19, modeled through the application of various transmission rates (0.001, 0.1, 0.15, 0.3, 0.5, 1.0, 1.5, 2.0, 2.3) as one of the inputs into the models. The achieved results are within the margin of error of +/−5% on the testing dataset. Khan and Gupta [89] applied the univariate time series model to predict the future number of confirmed cases in India. The authors applied an Auto-Regressive Integrated Moving Average (ARIMA) model to predict the data in the period from 26 March to 4 April 2020. The model has been trained using data from 31 January 2020 to 25 March 2020. The accuracy of predicted models has been validated using a nonlinear auto-regressive (NAR) network. The achieved results have shown high precision, and were evaluated using $R^{2}$, and have shown good prediction rates for 50 days without the need for adjustments by the researchers. The utilized Bayesian Information Criteria (BIC) values-based ARIMA (1,1,0) model achieved the $R^{2}$ value of 0.95, while the selected NAR model achieved $R^{2}$ values of 0.97 and constituted of 10 neurons trained with the Levenberg–Marquardt optimization algorithm. Kolozsvari et al. [90] applied the Recurrent Neural Networks (RNN) on the official data, provided by the government and collected from the datasets of JHU and the WHO. The RNNs are used with gated, recurring, Long Short-Term Memory (LSTM) units for the creation of two prediction models. The used RNN consists of the fully connected (dense) layer with the regression output layer used to determine the net value in the predicted time-series. The results are evaluated using root mean squared logarithmic errors (RMSLE). The evaluations are performed for Italy, UK, the US, Spain, France, Germany, and Hungary with RMSLE being below 0.5 for all countries using the first prediction model, and only models for France and the USA being above that value for the second prediction model. Tamand et al. [91] utilized the ANN to predict the time-series data for the number of confirmed and deceased cases in the USA, UK, France, China, South Korea, and India. The authors compare the achieved models and determine the possible future growth of cases in countries of France, the USA, the UK, and India based on the trends displayed in China and South Korea—which were ahead of the infection curve in the modeled countries. The authors predicted growth in all the countries based on both models used. Research presented by Direkoglu et al. [92] dealt with a 10-day forecast predicting the spread of COVID-19 based on a regional and global approach. A study predicting the spread of COVID-19 conducted on data for China, the Middle East, and Europe was presented. Furthermore, a prediction of global disease progression was also performed. An ANN architecture consisting of a single Long Short-Term Memory (LSTM) layer, a single dropout layer, and multiple fully connected layers was used to predict the spread of SARS-CoV-2 virus infection. Metrics based on RMSE were used to evaluate the quality of the prediction. The prediction, which stretched for 10 days into the future, was performed using selected networks trained for each region (or global model) separately. Networks were selected based on the RMSE value achieved in the last three days. Predictions showed a significant deviation from the actual data, where the prediction for the last day showed as much as 20% higher number of infected, but accurately predicted the trend of increasing global cases. The prediction of the number of deceased patients globally showed significantly better performance and minimal deviations.

Chimmula and Zhang [93] have proposed an LSTM-based approach for forecasting COVID-19 transmission. LSTM was trained by using data available until 31 March. The authors have concluded that the outbreak will end by the end of June. Considering the now available data, it can be noticed that such an assumption was accurate for the first wave of COVID-19 spread. Arora et al. [94] demonstrate the use of Deep LSTM, Convolutional LSTM (Conv-LSTM), and Bi-directional LSTM (Bi-LSTM) networks for predicting the number of COVID-19 cases in India. Since the Deep LSTM network, also known as the stacked LSTM network, has multiple hidden layers with multiple LSTM cells, it is considered as the extension of the conventional LSTM. By stacking multiple hidden layers, the depth of the ANN also increases, allowing the model to learn more complex sequences of the input data. In the case of the Conv-LSTM network, instead of using the multiplication function, the convolution operation is used in state transition. With Bi-LSTM, more complex time dynamics can be successfully modeled. As a performance measure, the mean absolute percentage error (MAPE) was used. According to the experimental results, the Bi-LSTM model achieves high accuracy for short-term prediction. Furthermore, an error value less than 8% was achieved for weekly predictions while an error value less than 3% was achieved for daily predictions. Chatterjee et al. [95] use several univariate Long Short-Term Memory (LSTM) models to forecast COVID-19 new cases and resulting deaths. The dataset used for univariate time-series forecasting was obtained from the “ourworldindata.org” website and it contains the number of total cases, death, and recoveries for the period from 1 January 2020, until 22 April 2020. Additionally, simulated datasets were used for correlation analysis and for analyzing the proposed algorithm. According to the experimental results, vanilla, stacked, and bidirectional LSTM models outperformed traditional multilayer LSTM models in terms of performance measures. Hartono proposes an LSTM-based method as a transmission predictor of COVID-19 disease which only requires the transmission similarities between countries as inputs [96]. Firstly, a transmission dynamics map was generated by utilizing a topological ANN. On the generated map, transmission similarities and dissimilarities between countries can be observed and a reference country can be chosen. After selecting the reference country, its longer dynamics can be used to train the LSTM model. With such an approach, transmission dynamics in a target country, which has similar dynamics as the reference country but shorter time series, can be predicted. Performed experiments show satisfactory values of performance measure in terms of three days predictions. To predict the number of COVID-19 confirmed and death cases, Aldhyani et al. [97] utilize a Long Short-Term Memory (LSTM) network and Holt-trend model. Data for the research were collected from the WHO. After the model is trained, three countries (Saudi Arabia, Spain, and Italy) are used for testing purposes. Various model evaluation criteria are used to evaluate the performance of the proposed models. As the results demonstrate, LSTM and Holt-trend models achieve effective performance in terms of predicting COVID-19 confirmed and death cases.

Yudistira [98] demonstrates the use of big data and the Long Short-Term Memory method to learn the correlation of COVID-19 growth rate. As input data, 100 regions (countries/provinces/states) are used for the model training process, while the other 4 countries (Indonesia, Sweden, Saudi Arabia, and Argentina) are used as validation data. Models were trained with the data collected from 22 January 2020, until 1 May 2020. The optimal structure of the models was determined heuristically. For model performance evaluation purposes, the mean squared error and RMSE are utilized. Experimental results show that LSTM outperformed RNN in terms of the RMSE value, therefore it can be used to predict COVID-19 spread. To predict the number of COVID-19 confirmed cases, Vadyala et al. [99] use a combination of LSTM networks, XGBoost, and K-Means. The presented approach is based on combining features of similar days to build an efficient model to forecast COVID-19 cases in Louisiana, USA. Data of COVID-19 cases were collected from the Center for Systems Science and Engineering at JHU, demographic data for the state of Louisiana was obtained from the Louisiana Demographic website, and the weather data were obtained from the National Weather Service website. As a result, the K-Means-LSTM method achieves satisfactory forecasting performance with the RMSE value of 601.20. Ayyoubzadeh et al. [100] use Long Short-Term Memory and linear regression models to estimate the number of positive COVID-19 cases in Iran. The data used in the research were obtained from the Google Trends and Worldometer websites. To achieve robustness of the models, 10-fold cross-validation is utilized, and as performance evaluation criteria, RMSE is used. According to the experimental results, the linear regression model achieves an RMSE value of 7.562 while the LSTM model achieves the RMSE value of 27.187. With more training data, authors believe that the LSTM model can achieve more precise predictions and outperform other ML-based models. Pal et al. [101] propose a shallow Long Short-Term Memory network. As model input data, the number of COVID-19 confirmed, recovered and death cases are used along with the weather data for a specific country. Additionally, a Bayesian optimization framework was performed to optimize country-specific networks. According to the results where the data of 180 countries are used as input, the proposed method outperforms state-of-the-art methods. In the case where a combination of the trend data and weather data are used together as model input, experiments show that the weather data do not have a significant impact on the model predictions. Zhao et al. [102] present curve fitting and various recurrent ANNs, including LSTMs and 10 different types of slim LSTMs to forecast the spread of COVID-19 in the USA. Dataset used to model the spread is publicly available and obtained from the JHU Coronavirus Resource Center. According to the presented results, LSTM RNNs tend to overfit, therefore, to fit the true distribution of COVID-19 input data, curve fitting is a better choice. Additionally, in terms of forecasting the pandemic, LSTM RNNs do not show a significant advantage over the curve fitting. ARIMA along with Nonlinear Autoregression Neural Network (NARNN) and LSTM was used in Kijrbas et al. [103] research for modeling confirmed COVID-19 cases. NARNN is used for time series predictions where an ANN utilizes a certain part of the time series as training data. LSTM is an ML algorithm with RNN architecture. The dataset was obtained from European Center for Disease Prevention and Control and consists of cumulative confirmed case data of eight different countries: Belgium, Denmark, France, Finland, Germany, Switzerland, Turkey, and the United Kingdom. In this research, seven performance metrics were used to identify mathematical differences and fairly besides graphical comparison. From the results, it can be seen that the LSTM model is the most successful for all country data examined.

Dutta [104], in his research, uses LSTM for predicting the trend of COVID-19 cases and fatalities. The dataset used in this research is publicly available [105] and consists of the global information regarding the COVID-19 infectious and deaths, the main focus is on the data from Michigan State. Since this is a time series problem, data are firstly pre-processed then converted into a form that fits the LSTM network. The performance of three LSTM models with different learning rates and activation functions are compared. The results show that the model with learning rate $1.2\cdot 10^{-3}$ and linear activation function is the best for the total case prediction while a model with learning rate $1.2\cdot 10^{-4}$ and linear activation function proves to be the best for total fatalities prediction. Tian et al. [106] show the use of three ML models including LSTM, Markov Chain model, and Hierarchical Bayes model for COVID-19 case prediction. The authors gathered a dataset from the Official JHU COVID GitHub repository for six countries (Germany, Italy, US, Taiwan, Japan, and South Korea) and compare different model’s performance for each country. LSTM in general proved to be an accurate model in predicting the epidemic trajectory for all selected countries including Germany, Italy, the US, and South Korea, but the Hierarchical Bayes model performs better than LSTM for Taiwan and Japan. Markov Chain model performs the worst for most of the countries and RMSE value varied greatly over different runs. Yan et al. [107] propose an improved LSTM-based method for predicting COVID-19 confirmed cases. As input data, authors use the 21-day case data of various regions and countries. Since the regions with a large number of cases can cause biased results of the model, the main idea behind the proposed improved method is to use a standard deviation of the last n days to adjust the parameters for the number of confirmed cases. Obtained experimental results show that the improved model has a better fitting effect along with a smaller prediction deviation.

In the research published by Pirouz et al. [108] is a case study of model construction for confirmed cases in Hubei, China. In this research, parameters such as:

- maximal daily temperature,
- minimal daily temperature,
- average daily temperature,
- population density of a city, and
- wind speed

were used for constructing the input vector. On the other hand, the output is defined as several confirmed cases. The dataset was constructed over 30 days. Such an approach was based on the integration of two ML methodologies: binary classification and regression. The binary classification was performed by using an algorithm called the Group method of Data Handling (GMDH). Classification is performed in such a way that an incidence is labeled with 0 if the number of confirmed cases does not exceed 850. On the other hand, regions with an incidence higher than 850 are labeled with 1. By using the presented methodology the highest accuracy of 95.7% is achieved. When regression analysis was used, $R^{2}$ up to 0.65 was achieved. The authors have concluded that these results have confirmed the possibility of utilization of environmental parameters for modeling the spread of infectious diseases. Javid et al. [109] have proposed a predictive analysis based on a single-layer ANN called extreme learning machine (ELM) for estimation of COVID-19 spread in 12 world countries. ELM is trained by using data provided by JHU for Sweden, Denmark, Finland, Norway, France, Italy, Spain, UK, China, India, Iran, and the USA. To determine the quality of designed predictors, different time intervals were used for ELM training. The predictions were performed for the next 14 days, starting from the day after the last date included in the training data. To achieve higher prediction performances, a sliding window approach is utilized. The proposed system has enabled high performances for all countries included in this study. Huang et al. [110] have proposed a CNN-based approach for estimation of COVID-19 cases in Chinese provinces and cities. Alongside CNN, methods based on Gate Recurrent Unit (GRU), LSTM, and MLP were used as well. The CNN is trained and tested by using data available for cities: Wuhan, Huanggang, Xiaogan, Ezhou, Yichang, Wenzhou, and Shenzhen. From the obtained results it can be seen that the lowest error rates were achieved when CNN is used, regardless of input layer configuration. Furthermore, it can be noticed that the highest error rates were achieved with MLP followed by LSTM and GRU. The research presented by Haghshenas et al. [111] was based on AI utilization to determine the influence of environmental parameters such as population density of each region, average daily temperature, relative humidity, and wind speed on the number of the positive cases in the following days. The analysis was performed in the period between 14 February 2020 and 24 March 2020. The conducted study used data collected in four Italian regions of Lombardy, Veneto, Piedmont, and Emilia-Romagna. The measurable data were collected in major cities of each region: Milan for Lombardy, Venice for Veneto, Turin for Piedmont, and Bolonia for Emilia-Romagna. Collected data were used to train the ANN-based estimator. To determine the optimal hyperparameters for the proposed ANN, Particle Swarm Optimization (PSO) algorithm and differential evolution (DE) algorithm were utilized. From the presented results, it can be concluded that both approaches achieved similar results from the standpoint of estimation quality. On the other hand, the PSO-based approach has achieved the desired estimation performances in a lower number of iterations. In the end, the authors have concluded that relative humidity, among other environmental parameters, has the highest impact on COVID-19 spread dynamics.

Same as in the previous section, the comparison for similar algorithms, in this case RNN-based ones, are given in Table 6—for those algorithms which had the results evaluated numerically.

### 4.4. Other Papers

Dal Molin Ribeiro et al. [112] compared the various methods for determining the regressive models for the spread of COVID-19. In addition to ARIMA, which was previously used in other research Dal Molin Ribiero and their coauthors used cubist regression (CUBIST), random forest (RF), ridge regression (RIDGE), and support vector regression (SVR). The models obtained from the above methods are utilized as meta-learners, with Gaussian Learning Process (GLP) used as a meta-learner in the stacking-ensemble meta-learning approach. The best results have been achieved by the SVR and stacking ensemble learning processes with the lowest errors achieved being 0.87% for the prediction period of one day, 1.02% for the prediction period of three, and 0.95% for the prediction period of six days. Vaid et al. [113] demonstrated the utilization of Bayesian susceptible-infected-recovered (SIR). Kalman filter and ML techniques to accurately forecast future occurrences of COVID-19 cases across countries with different anti-covid policies. The comparison is made between countries with relaxed policies, such as Sweden, and stringent policies—such as the USA. Multiple insights are discovered. The authors determined that the change in spread rates is apparent when the policies are implemented. The drop in the new infection rate is sharper in the case of rigorous policies being applied, with a more gradual drop shown in the case of lighter rules being implemented. Authors predict a downward trend in countries with stricter policies and upward one in those with relaxed ones—concluding that the stricter policy implementations greatly assist with the lowering of COVID-19 infection rates. Tuli et al. [114] apply cloud computing data collection, with ML techniques to track the epidemiological spread of COVID-19 and predict the future spread. Authors apply the iterative weighting process to fit the Generalized Inverse Weibull distribution, which achieves a quality enough fit for the method to be used as a base of the cloud framework the authors propose. The framework in question can obtain data from government centers and private hospitals to combine the positive patient numbers with other data such as population density, average and median age, weather conditions, quality of health facilities, and others. Such a framework can be implemented using existing cloud services such as Azure, with a flexible computing cost—starting at just 1.2 USD a day, but predicted to increase with the dataset size. Melin et al. [115] demonstrated the utilization of an unsupervised ANN, the so-called self-organizing map method in the prediction of the spatial spread of the COVID-19. Unlike the previously proposed methodologies, this method allows the authors to observe which countries cluster together—indicating a similar behavior. The spread is modeled by the authors through the observation of the spatial dimension of the spread modeling, unlike the previous papers which used the temporal dimension of the COVID-19 spread to model and predict the spread of the disease in the future. The importance of this approach lies in the application of similar strategies in the countries in which the behavior of the virus showed similarities, allowing for successful strategies to be reapplied.

Kapoor et al. [116] have performed modeling for coronavirus forecasting using spatio-temporal graph ANNs. The authors used the so-called Graph Neural Networks (GNNs) with the mobility data. Nodes in the GNN network represent the regional human mobility, while the connecting spatial edges represent the inter-regional mobility rates and temporal edges represent the node feature change in time. The approach is evaluated on the county level inside the US. In comparison with the existing baseline models, the authors, using the proposed approach, managed to lower the RMSLE by 6% and increase the absolute Pearson Correlation improvement from 0.9978 to 0.998. The research presented by Rustam et al. [117] used four standard regression techniques: linear regression (LR), least absolute shrinkage and selection operator (LASSO), support vector machine (SVM), and exponential smoothing (ES). Each approach was used to estimate the number of newly infected cases, number of deceased patients, and number of recovered patients. The results have shown that the highest performances were achieved if ES was used, with $R^{2}$ scores of 0.98, 0.97, 0.99 achieved for estimation of infected, deceased, and recovered patients, respectively. On the other hand, it can be noticed that by using other approaches, significantly weaker results were achieved, with an exception of LASSO for estimation of infected and deceased patients, with achieved $R^{2}$ scores of 0.98 and 0.85 respectively.

Ponia et al. [118] have used the exponential smoothing method and autoregressive integrated moving average (ARIMA) to forecast 10-day COVID-19 spread dynamics across Indian states. The proposed method is trained by using data from 30 January and 21 April and used to forecast spread dynamics until 1 May. Khan and Hossain [119] apply ML techniques on the problem of determining covariates with high importance to the cumulative number of confirmed cases. Researchers apply RT, cluster analysis, and principal component analysis on the data of 133 countries obtained using the Worldometer website up to the 17 April of 2020. The research indicates clustering of countries based on the analyzed data, with 4 clusters emerging. The authors conclude that for the prediction of the number of cumulative cases, the number of tests per country is not an important variable. To estimate the evolution of COVID-19 in Spain, Cabras et al. [120] use a combination of modern Deep Learning techniques along with the Bayesian Poisson-Gamma model. The database used in this research is publicly available and obtained from Instituto Carlos III de Madrid in Madrid [41]. As a Deep Learning technique, a bidirectional LSTM network is imposed whose output is afterward processed with a Bayesian Poisson-Gamma model. As a result, such a hybrid approach can be used to predict the evolution of the pandemic as well as to estimate the consequences of eventual future scenarios. Ndiaye et al. [121] propose an ML and SIR modeling, using deterministic and stochastic cases, with the numerical approximation to predict the number of patients infected with COVID-19 in periods ranging from few days to three weeks. While the presented results show good fitness with the existing data, the authors incorrectly predict the end of the pandemic no later than the beginning of May 2020, which is apparently an incorrect prediction. To predict the number of COVID-19 confirmed cases daily, Yahia et al. [122] propose a deep ensemble learning method. Such an ensemble consists of deep ANNs, Long Short-Term Memory networks, and Convolutional ANNs, thereby, the advantage of each algorithm can be used to improve forecasting results. Three experimental scenarios were used to validate the robustness and effectiveness of the proposed method. As the model performance measures, RMSE and accuracy are utilized. Experimental results show that the accuracy of the stacking method can be improved by fusing the forecasted values of DNN, LSTM, and CNN. Wang et al. [123] use a logistic model and ML-based model to predict the epidemic trends in COVID-19. Research data related to COVID-19 were obtained from JHU for the time from 22 January 2020 to 16 June 2020. Additionally, the 2003 SARS epidemic data were obtained from news-site (SOHU). First, the logistic model is used to fit the cap of the epidemic trend, and afterward, the cap value is used as FbProphet model input to model the epidemic curves and predict the trend of the COVID-19 pandemic. According to the experimental results, the global outbreak peak was expected in late October with the estimation of 14.12 million infected people.

Onovo et al. [124] used Supervised ML and Empirical Bayesian Kriging (EBK) techniques to reveal correlates and patterns of COVID-19 disease outbreaks in sub-Saharan Africa. EBK is a geostatistical interpolation method where parameters are automatically calculated through a process of subletting and simulations. The dataset used for this research was obtained from JHUand it consists of time series data on the outbreak of COVID-19 across sub-Saharan Africa. For statistical inference and variable selection, they used LASSO. The obtained results show that doubling time in new coronavirus cases was 3 days. Churasia and Pal [125] demonstrate several forecasting techniques to predict the future spread of COVID-19. The naive method, moving average, simple average, single exponential smoothing, Holt-Winter method, Holt-linear method, and ARIMA are compared to improve the RMSE score. Dataset used in this research was obtained from the World Health Organization and consists of information about the observation date, state, country, and latest updates. The best model for time series data over all other methods was the ARIMA model. ARIMA is a combination of a differenced autoregressive model with the moving average model and it shows that time series is regressed on its past data. The results show that the number of deaths will increase by more than 600,000 by January 2021 and beyond. Stochitoiu et al. [126] demonstrate the model which can predict the number of daily fatalities in Romania for up to three weeks in the future. The obtained results show that the fatality rate is notably smaller (≈0.3%) than the worldwide average. Based on the publicly available dataset they implement an optimized mathematical model based on SEIR for estimating the evolution of COVID-19. Susceptible-exposed-infectious-removed (SEIR) is a standard method for modeling the evaluation of infectious diseases. In this research, the authors optimize the parameters of the model where the Convolutional Neural Network (CNN) learns from synthetic data produced by Modified SEIR to predict the correct parameter set. By integrating an improved adaptive neuro-fuzzy inference system (ANFIS) and enhances flower pollination algorithm (FPA) along with the Salp Swarm algorithm (SSA) Al-Qaness et al. [127] achieved satisfactory results. The proposed model called FPASSA-ANFIS forecasts and estimates the number of confirmed COVID-19 cases in the upcoming 10 days. FPASSA-ANFIS starts by formatting input data into time-series form, and it utilizes an improved FPA algorithm to train the ANFIS model. Dataset used in this research is publicly available and obtained from the WHO website, where 75% of data is used as a training set and 25% as a test set. As evaluation data authors used two datasets of weekly influenza confirmed cases in the USA and China obtained from Centers for Disease Control and Prevention (CDC) and the WHO website. By analyzing the results of MAE, MAPE, RMSE, RMSRE, and CPU time, it can be concluded that the proposed method outperforms other models with an $R^{2}$ value of 0.97. Maliki et al. [128] demonstrate the use of ML algorithms to extract the relationship between different factors and COVID-19 spread rate, more specifically to estimate the impact of weather variables on the transmission of coronavirus. The dataset was obtained from JHU Center for System Science. Weather variables used in this research are temperature and humidity. From the obtained results it can be concluded that, in the case of death rate, weather variables are more important than variables such as urban percentage or age. Gupta and Gharehgozli [129] as well study the impact of weather variables, along with social and demographic variables on the spread of COVID-19 in the US. The weather (temperature and humidity) dataset was obtained from AirNow, while the dataset for pollution was obtained from the Iowa Environmental Mesonet and the dataset for per capita GPD was obtained from the Bureau of Economic Analysis. ML models used in this research are linear regression, support vector machine (SVM), and DT. From the obtained results it can be seen that air pollution, higher temperature, and population density have a positive impact on the spread, while the per capita GDP has a negative effect.

In their research, Velásquez and Lara [130] use Reduced-Space Gaussian Process Regression to forecast the spread of COVID-19 in the USA. The dataset was obtained from the Center for Systems Science and Engineering at JHU. Gaussian Process Regression (GPR) is nonparametric and works well on small datasets. In this research, it is used with application in dynamical and chaotic systems. Using the proposed model, they predicted that the epidemic would reach saturation in July 2020 in the USA. In addition, with new restrictions and quarantine implemented in the USA, the number of new coronavirus cases could stagnate, but in the next two months, it could generate a critical rate of new COVID-19 cases and deaths. Uhlig et al. [131] use AI algorithms to provide epidemic forecast and risk calculations for outbreaks. Time-series data of China, Japan, South Korea, the US, Russia, Germany, Italy, Spain, and the UK is used in this research and it was obtained from the Center for Systems Science and Engineering (CSSE) and other aggregator websites. Results show a correlation between confirmed case numbers and real-time change in the effective reproduction number. In the case of South Korea, the model predicts local outbreaks while in the case of Germany and the US it predicts a gradual decrease in epidemic potential in the ensuing days. Pasavat et al. [132] in their research use two different models, mathematical and ML, for predicting the positive cases, with concern to lockdown. As an ML model, they use linear regression to predict the number of positive cases in India if lockdown continues. In linear regression, the relationship between explanatory and dependent variables follows through a line that usually represents the relationship between two variables. The dataset used in this research is publicly available and collected from Humanitarian [133]. It consists of a day-wise number of cases, recovered, and deaths for India. Results show that in different states of India positive cases are rapidly increasing, with an $R^{2}$ score of 0.9078. Al-Qaness et al. [134] shows the improved version of the adaptive neuro-fuzzy inference model (ANFIS) to forecast the number of infected people in the US, Korea, Iran, and Italy. For optimization of ANFIS, a new nature-inspired optimizer, Marine Predators algorithm (MPA) is used. The Marine Predators algorithm is a newly nature-inspired optimizer, and it is very similar to other metaheuristic techniques. The MPA depends on the survival of the fittest strategy and it has been selected for predators for surviving. Just like in other metaheuristic algorithms, the initial solution of the MP algorithm is uniformly distributed in the search space. According to the results of the testing set, the $R^{2}$ of the proposed model is 0.9595, 0.9648, 0.9874, and 0.9859, for the USA, Korea, Iran, and Italy, respectively. Abhari et al. [135] demonstrate the use of an agent-based AI simulation platform, called EnerPol to predict the evolution of COVID-19 in Switzerland. In the EnerPol model, agents adapt their behavior through AI. Data used in this research are publicly available from JHU and adapted to Swiss demographics. From the obtained results, it is shown that without social adjustments and government interventions, the explosive spread of the COVID-19 virus and the number of infected people reaches 42.7% of the total population of Switzerland by 25 April 2020. Erraissi et al. [136] use an ML model to predict the number of infected COVID-19 cases in Morocco. Research is realized in Spark ML with the ’Scala’ language and tested for a certain number of algorithms. The classification algorithms used are SVM, random forest (RF), logistic regression, decision tree (DT), voting classifier (VC), and Gaussian Naïve Bayes (NBC). The authors collected data from the site of the Moroccan Ministry of Health. Results show that the proposed method achieves satisfactory results, and it can be applied to data for all countries in the world. Carrillo-Larco and Castillo-Cara [137] use unsupervised ML algorithms to cluster countries in groups with shared profiles of coronavirus pandemic. Parameters that were used are metrics of air pollution, health system coverage, socioeconomic status, and disease prevalence estimates. Different data sources are collected to build a unique dataset with information on COVID-19. The authors used a one-way ANOVA test and compared the clusters in terms of the number of confirmed cases, the number of deaths, the case fatality rate, and when the first case of COVID-19 appeared. From the obtained results it can be concluded that the model with three principal component analysis (PCA) parameters and five or six clusters showed the best capability to group countries in relevant sets.

## 5. Modeling of COVID-19 Using EC Methods

In addition to various ML algorithms, there have been numerous implementations of EC algorithms to develop epidemiology models of COVID-19 diseases spread. EC algorithms are algorithms based on the principle of evolution, with the principal algorithm being the genetic algorithm (GA). GA and its derivatives are based on the creation of several possible solutions, and application of mechanisms such as crossover (creation of new solution from two candidate solutions that fit the problem well) and mutation (random modification of solutions) to raise their fitness to the problem, and achieve high-quality solutions. The application of this type of algorithms in the modeling of COVID-19 epidemiological spread will be provided in this section. Niazkar et al. [138] have implemented multi-gene Genetic Programming (GP) to predict the number of confirmed cases for China, the Republic of Korea, Japan, Italy, Singapore, Iran, and the USA in a period from 15 March up to 5 April 2020. GP has also been used by Salgotra et al. [139] to develop prediction models for confirmed cases and death cases for the Indian three most affected states at the time Maharashtra, Gujarat, and Dehli as well as the whole of India. The results showed that these models are highly reliable for the time series prediction of COVID-19 cases in India. The same authors [140] have used GP to mathematically model the potential effect of coronavirus in the 15 most affected countries of the world. Two datasets of confirmed cases and deceased cases were taken into consideration to estimate, how transmission varied in these countries between January 2020 and May 2020. The proposed model predicted that the transmission of COVID-19 in China is declining since late March 2020. In Singapore, France, Italy, Germany, and Spain the curve has stagnated. In the case of Canada, South Africa, Iran, and Turkey the number of cases is slowly increasing. However, in the case of the USA, UK, Brazil, Russia, and Mexico the rate of increase is very high and control measures need to be taken to stop the chains of transmission. The GA was used by Rayungsari et al. [141], for the estimation of parameters in the generalized Richards model by adjusting COVID-19 case data in Indonesia. The dataset consisted of daily new cases and a cumulative number of COVID-19 cases in Indonesia in the period from early March to early June 2020. The best parameters in the generalized Richards model were chosen based on the lowest cost function value, determined from the distance between data with estimated model and real data. The results obtained in this study are not entirely consistent with the real data. The numerical simulations also showed that daily new cases would reach the peak in early June 2020, with around 600 cases per day, and would stop in the middle of February 2021 with a maximum cumulative amount of 65,067. However, this prediction was apparently mistaken. Rabbah et al. [142] have implemented a genetic fitting algorithm and cross-validation method to obtain a mathematical epidemic model to study COVID-19 outbreak dynamics of Algeria in the period between 25 February and 24 May 2020. In this study, the cross-validation method was used to overcome the overfitting problem. The results showed that the basic reproduction number is estimated to 3.78 (95% confidence interval 3.033–4.53) and the effective reproduction number on May 24th after three months of the outbreak is estimated to 0.651 (95% confidence interval 0.539–0.761). In research performed by Yousefpour et al. [143], the mathematical model for SARS-CoV-2 transmission based on Wuhan’s data was developed using a multi-objective genetic algorithm. To solve the problem effectively this algorithm was used to achieve high-quality schedules for various factors including contact rate and transition rate of symptomatic infected individuals to the quarantined infected class. This investigation demonstrated that by applying the proposed optimal policies, governments could find useful and practical ways to control the disease.

The combination of the virus optimization algorithm (VOA) and ANFIS was used by Behnood et al. [144], to investigate the effects of various climate-related factors and population density on the spread of COVID-19. In this study, the data on the climate-related factors and the confirmed infected cases by the COVID-10 across the US countries was used. In this investigation, the population density had the most significant impact on the performance of the developed models, which indicates the importance of social distancing in the reduction of infection rate and spread rate of the COVID-19. The increase of maximum temperature was found to slightly reduce the infection rate. Other factors such as average temperature, minimum temperature, precipitation, and average wind speed were insignificant to the spread of COVID-19. However, the investigation showed that a slight increase in the relative humidity has slightly increased the infection rate. The GP was applied by Howard [145] to a visitation scheduling solution that can deliver a less austere COVID-19 pandemic population lockdown. In this investigation, a novel partial infection model is introduced to discuss the proof of concept solutions which are compared to round-robin uninformed time scheduling for visits to places. The computations indicate vast improvements with far fewer dead and hospitalized. Ghosh and Bhattacharya [146] have used the probabilistic cellular automata-based method to model the infection dynamics for a significant number of different countries. This study showed that for accurate data-driven modeling of this infection spread, cellular automata provide an excellent platform, with a sequential genetic algorithm for efficiently estimating the parameters of the dynamic. The results demonstrated that the proposed methodology is flexible and robust at the same time and can be used to model the daily active cases, the total number of infected people, and total death cases through systematic parameter estimation. Hosseini et al. [147] demonstrate an application of COVID-19 distribution process modeling and control through the use of a novel COVID-19 optimizer algorithm. Authors simulate the COVID-19 spread in multiple infected countries and model the distribution as a process. Optimization is performed to minimize the number of affected countries. The results show that the proposed algorithm provides better results in the comparison with Volcano Eruption Algorithm, Gray Wolf Optimizer, Particle Swarm Optimization, and genetic algorithm, proving the needs for its application. The COVID-19 epidemic transmission via $SEIAS_{q}E_{q}HR$ paradigm was formulated by Higazy and Alyami [148], using the Caputo-Fabrizio fractional derivation method. In the suggested fractional-order COVID-19 $SEIAS_{q}E_{q}HR$ paradigm, the impact of changing quarantining and contact rates are examined. The stability of the proposed fractional-order paradigm is studied and a parametric rule for the fundamental reproduction number formula is given while the existence and uniqueness of the stable solution are proved. The genetic algorithm was used to perform an optimum control strategy, and the peak values of the infected population classes are minimized. The results of the conducted investigation showed that the proposed fractional model is epidemiologically well-posed and is a proper choice.

Elmousalami and Hassanien [149] have performed comparison models on COVID-19 affected cases using time series models and mathematical formulations. This study presents a comparison of day-level forecasting models on COVID-19 affected cases using time series models and mathematical formulation. The forecasting models and data strongly suggest that the number of COVID-19 cases grows exponentially in countries that do not mandate quarantines, restrictions on travel, and public gatherings. The development of the appropriate model that captures the COVID-19 data from South Korea and the exploration of the non-linear behavior was performed using a genetic algorithm, as reported by Kwuimy et al. [150]. The authors have used the nonlinear susceptibl-exposed-infectious-removed transmission model with added behavioral and government policy dynamics. The genetic algorithm was used to identify key model parameters. The parametric analysis revealed conditions for sustained epidemic equilibria. The obtained results showed that the nonlinear dynamic analysis in pandemic modeling demonstrates the dramatic influence of social government behavior on disease dynamics. The modeling of the epidemiology curve for China, Italy, Spain, the USA, and as well on the global scale in time from 22 January up to 8 April of 2020 was performed using genetic programming as reported by Anđelić et al. [151]. To obtain the mathematical model for estimation of epidemiology curve for countries and on the global scale first the mathematical model for estimation of the number of confirmed, deceased, and recovered cases for each country and on a global scale was obtained. The initial data for the development of each mathematical model for estimation of the number of confirmed/deceased/recovered cases of each country, consisted of latitude and longitude values of central locations of the specific country and the number of days since the outbreak began, while the output value was the number of confirmed/deceased/recovered cases, respectively. In the case of a mathematical model for estimation of the number of confirmed/deceased/recovered cases on a global scale, the input value was the number of days since the outbreak began while the output value was the number of confirmed/deceased/recovered cases. All obtained mathematical models were tested on the testing portion of the dataset to measure the correlation coefficient value. The conducted investigation shows that the best mathematical models produced for confirmed and deceased cases achieved $R^{2}$ scores of 0.999, while the models developed for estimation of recovered cases achieved the $R^{2}$ score of 0.998. The equations generated for confirmed, deceased, and recovered cases were combined to estimate the epidemiology curve of specific countries and on the global scale. The estimated epidemiology curve for each country obtained from these equations is almost identical to the real data contained with the dataset. A hybrid approach is demonstrated by Ardabili et al. [152] who applied a Gray Wolf Optimizer (GWO) algorithm to optimize the weights of a Feed Forward (FF) ANN. The authors applied the proposed methodology on a global dataset and have evaluated the achieved models using MAPE, achieving an error of 11.4% on the validation dataset. Another application of GP was reported by Anđelić et al. [153] for estimation of the epidemiology curve of the entire US during 22 January up to 3 December 2020. In this investigation, only 50 federal states were considered. For each federal state, the mathematical expressions for estimation of the number of confirmed/deceased/recovered patients were obtained on the training dataset while the testing dataset was used to evaluate obtained mathematical expressions and to calculate the $R^{2}$ correlation coefficient. The data for each federal state consisted of the central location of that federal state (latitude, and longitude), the number of days since the outbreak began (317 days), and the number of confirmed/deceased/recovered patients for each day since the outbreak started. After all mathematical expressions were obtained the mathematical expression for estimation of the number of confirmed/deceased/recovered patients for the entire US were formulated. Using these three mathematical expressions, the mathematical expression for estimation of epidemiology curve was obtained. The results showed that obtained mathematical expression for estimation of the epidemiology curve for the entire US achieved the $R^{2}$ score of 0.9933. The conducted investigation also showed that the GP algorithm can produce mathematical expressions for estimation of the number of confirmed, deceased, recovered patients as well as epidemiology curve not only for the federal-state but for the entire US with very high accuracy.

The results of selected papers are given in Table 7. As before, only those papers which used a metric as opposed to the visual estimation, and were focusing on similar goals, are displayed within the table.

## 6. Observations

**Figure 9:**
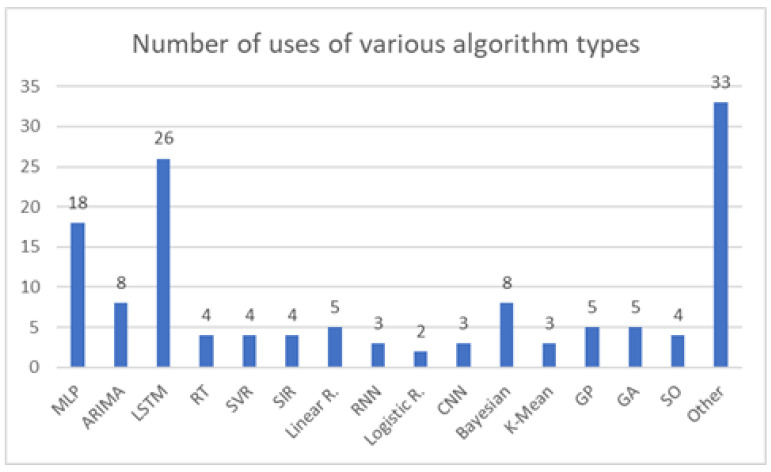
The number of uses of individual algorithms among the reviewed papers. Similar algorithms have been grouped. Abbreviation “R” signifies Regression, with other abbreviations given in the text. Single-use algorithms have been grouped into “Other”.

This section will briefly present the observations that we have noticed from the papers whose overview was provided in previous sections. First, we observed that ML algorithms enjoy much higher popularity among the researchers. Among the selected papers, 70 have used ML algorithms, with EC algorithms being used by 16 papers. This higher popularity may be caused by the wider availability of ready-to-use libraries for ML algorithms [154,155,156]. Among the reviewed papers most algorithms have used an already existing algorithm. In these cases, the research novelty was based on the application of said algorithms in various research goals related to the novel COVID-19 virus. Less than 10 of the reviewed papers offer algorithmic novelty. It is also noticeable that a large number of available research in the observed field is published as pre-prints. While research like that should be considered carefully, due to not passing a peer-review process, this trend demonstrates the importance of pre-print availability in such problematics as novel pandemics, in which the exchange of research results and ideas needs to happen rapidly. Many of the research items reviewed do not provide the trained models in an easy-to-use manner. While the replicability of results is indeed possible without them, training ML models may take a large amount of time. By publishing the results in an open-source repository in cases where the models generated are not easily displayable either due to size (larger RT models) or complexity (common concern with ANNs), or providing the models generated in the appendix of their work (in cases of algorithms such as GP), authors may achieve higher applicability of their results—an important concern in a pandemic which may require fast actions. Among the observed research, 39 items have used and compared more than one algorithm. Among them, some researchers have used the algorithms separately and compared results to determine a superior algorithm, but many have taken the hybrid approach—combining multiple algorithms to improve their performance. A popular trend in this space seems to be the application of EC algorithms on the problem of optimal hyperparameter search of the ML algorithms. From reviewed papers, it is obvious that some methods are more commonly used for the problem of epidemiology spread modeling. Figure 9 shows the comparison of times each separate method has been used. Similar algorithms with minor variations have been grouped for easier display.

**Figure 10:**
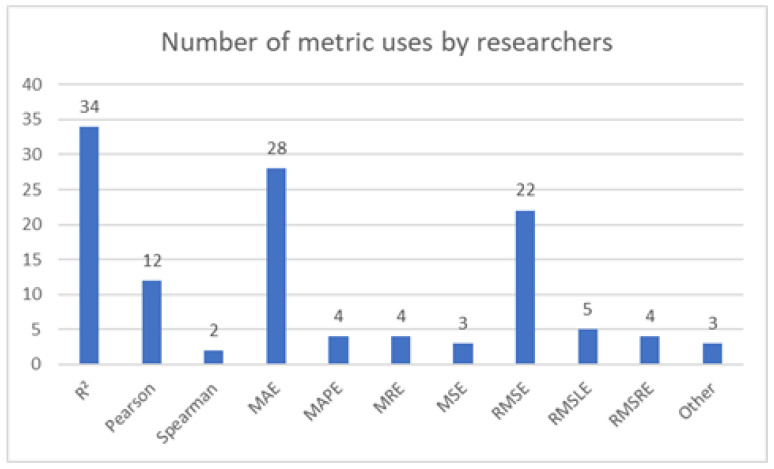
The number of metric uses within the reviewed papers. Abbreviations have been given in the text. Single-use metrics have been grouped into the “Other” category.

From Figure 9, it can be seen that the most popular type of method used in the reviewed papers was LSTM. As LSTM is commonly used for regression of time-series-based data, it is apparent why it was a popular choice [157,158]. Still, methods that do not take the time-series nature of data into account by their design have also shown high popularity, with MLP being the second most used method at 18 uses. MLP is followed by ARIMA and Bayesian-based regression methods. Other methods that have been used multiple times are also shown in Figure 9, while the methods which have been used only once have been grouped into the “Other” category. One important factor to note is also the metrics used by the researchers to determine the quality of their models. These metrics are displayed in Figure 10. By observing the metrics used, it can be seen that a high amount of researchers have opted to utilize multiple metrics—with a total of 76 observed researchers using more than one metric. In almost all of these papers, 74 of them, researchers have used at least one metric which evaluates the error (such as MAE, RMSE, or others) of the model, and another metric which evaluates the quality of the regression more directly (such as $R^{2}$ or Pearson coefficient) which is a standard and suggested practice when evaluating regression models [35].

As Figure 10 shows, the coefficient of determination $R^{2}$ is the most popular metric used. Since it evaluates the amount of variance between the real and predicted sets, it is a good metric to evaluate the quality of achieved regression as well as providing an easily interpretable result. Most researchers have paired it with an error metric, such as RMSE to quantify the quality of the model more easily with the numeric data. Other regression metrics that were used were Pearson and Spearman correlation coefficients, with Pearson being used much more often than Spearman, but still much more rarely than $R^{2}$. When it comes to error metrics MAE was the most popular metric used, with 28 researchers implementing it. RMSE was the second most used, with 22 researchers selecting it for model evaluation. Other variants of error metrics have been used sporadically. As was the case with methods used, metrics that have only been used once, such as Getis–Ord Gi* test, were grouped for the display.

**Figure 11:**
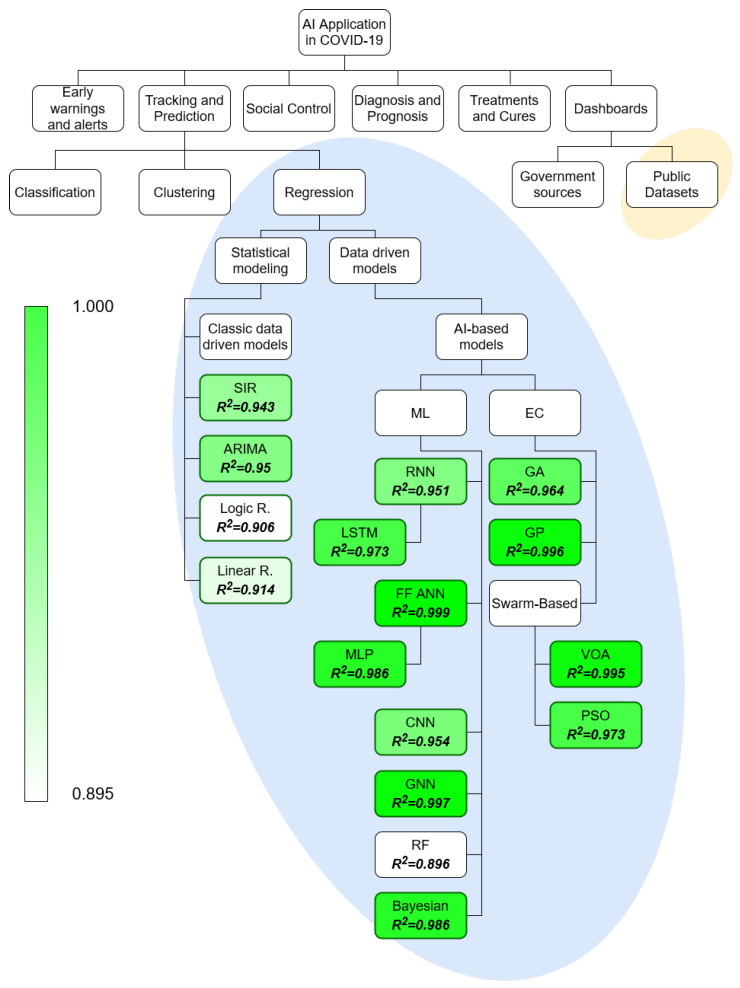
Taxonomy of the AI research in the field of COVID-19, with the best results for the reviewed research subjects given inside the taxonomy graph.

The comparison of the best results achieved is given in Figure 11. All reviewed types of algorithms are marked using a green outline. To allow for a direct comparison of algorithms, only those papers which attempted to determine a global number of confirmed cases, have been used. This criterion was selected because the particular goal has been addressed by most researchers. Further investigation showed that $R^{2}$ was the most popular metric amongst them, so it was selected as the evaluation criteria for the creation of visual algorithm performance comparison. Figure 11 shows that the overall best results are achieved using the Feed Forward ANN algorithm, while the poorest are achieved by the RF and logical regression algorithms. If we adopt that a regression score of $R^{2}>0.95$ is considered satisfactory [159,160], then all AI-based models except for Tree-based algorithms have achieved such a score. The value of the score is displayed numerically and with the background color (the value of color is given on the scale found on the left of the Figure 11).

## 7. Conclusions

Literature data provide strong evidence for the role of AI-based modeling of COVID-19 epidemiological spread. A high number of reviewed methodologies show great promise and were capable of obtaining high-quality models of epidemiological curves in the observed areas. Multiple trends arise among the research. The first noticeable trend is the application of existing ML and EC regression algorithms to regress COVID-19 epidemiological spread curves, as opposed to the development of custom, novel algorithms specific to the task. Many researchers apply both regression quality estimate metrics in combination with the error metrics to better quantify their results. The same is true for the combination of multiple algorithms—either comparing them or combining the algorithms to develop a hybrid model. As previously mentioned, most researchers utilized publicly available data, indicating extremely high importance in allowing open access to the data in order to develop potentially useful models. A large number of research items available online are preprints, still awaiting publication. This trend indicates the popularity of the utilization of preprint servers such as *Preprints*, *Arxiv*, and *Medrxiv* for state-of-the-art, hot-topic analyses.

As for the research questions posed in the introduction, the following conclusions can be drawn. Most researchers utilize the publicly available datasets, and it can be concluded that the wide availability of data enables a lot of research to be performed and published. EC algorithms have been applied to the problem of regressive COVID-19 spread modeling, with high precision results. The most commonly used algorithms in the COVID-19 spread have been LSTM when the regression is performed from the data formatted as time-series, and MLP when the data are formatted as a regression dataset. The evaluation was most commonly performed with two metrics—with the most common being $R^{2}$ and MAE. The results obtained from the preprints show that there is a large number of research items available in the aforementioned pre-print servers. Still, as shown in the methodology section using PRISMA, a large number of research items published in the preprint servers have been discarded and not included in the review because of the low quality of the pre-published manuscript, especially in the methodology, results, and conclusions of the research. While there are many high-quality items pre-published, and the early availability of such results is crucial for the situations as an ongoing pandemic, researchers should be careful when utilizing pre-published research and use their best judgment in determining the quality of published and peer-reviewed research of similar topic.

Collectively, all these findings clearly show that AI-based modeling of COVID-19 is of utmost interest to the global scientific community. Highly precise epidemiological spread models are achieved in many cases. This points towards the potential use of AI-based regression algorithms for epidemiological spread modeling and prediction in the future pandemics that may be faced in times to come.

## Figures and Tables

**Table 1:** Review methodology with explanations, according to PRISMA 2020 statement.

Item	Objective	Explanation
5	Eligibility criteria	Articles that were included in this review were chosen according to the inclusion and exclusion criteria. The main inclusion criteria were to only include publications that are thematically linked with the COVID-19 pandemic. Furthermore, only articles where an AI-based approach was used in COVID-19 spread modeling were included in this systematic review. Articles related to spreading modeling without AI application and articles where AI was used in other COVID-19 related problems (e.g., clinical problems) were excluded from the review. All included studies were sorted into two groups: studies related to ML and studies related to evolutionary algorithms.
6	Information sources	The search for studies that were included in this systematic review was performed through multiple scientific databases and registers. Due to the specificity of the COVID-19 pandemic as a relatively new scientific challenge, databases of scientific papers available in open access were used. Articles collected for this review were founded by searching through the following bases: Google scholar, The Multidisciplinary Preprint Platform, PubMed, Web of Science, Arxiv, and MedArxiv. All databases were searched between 15th December 2020 and 10th April 2021.
7	Search strategy	The search for publications was performed with search filters that assure Eligibility criteria explained by PRISMA item 5. Furthermore, only articles available in open access were included in this systematic review.
8	Selection process	Determination whether the article fits the inclusion criteria is performed by multiple reviewers. All screenings were independent and the minimal number of reviewers that have screened one article is three.
9	Data collection process	Data collected from all reports are collected from the results and conclusion sections of the articles, as well as abstracts. As stated in item 8, the minimal number of three reviewers was assigned to one article.
10a	Data items	All articles were evaluated according to regression measures used for evaluation of AI-based regressors (R2-score, Accuracy, MAE, RMSE).
10b		All articles that do not have quantifiable results are excluded. These criteria are derived from the assumption that articles without quantifiable results can not be compared with other studies.
11	Study risk of bias assessment	To access bias, during the reviewing process, at least three reviewers were assigned to one study. During the aforementioned process, the work of all reviewers was independent.
12	Effect measures	To unify the results of the reviewing process, only articles that have passed the criteria of all reviewers were included in the systematic review.
13a	Synthesis methods	To define the impact of AI methods used on regression performances, three different syntheses were performed. The first synthesis is related to the comparison of ANN-based methods. The second synthesis is performed of RNN-based regression methods. Finally, the third synthesis was performed to compare methods based on evolutionary algorithms.
13b		To synthesize the findings, all results were sorted according to three categories: the number of infected cases, the number of recovered patients, and the number of deceased patients.
13c		All three syntheses were concluded by tabulating the results according to the three described categories.
13d		Due to the multiplicity of metrics used in various studies, synthesis is performed as a qualitative comparison of achieved results. Such an approach was chosen due to the impossibility of performing a meta-analysis.
13e		Possible causes of heterogeneity among study results were not explored.
13f		To achieve the robustness of the performed syntheses, analysis of the distributions of the algorithms used were performed. Such an approach was used to prevent excessive deviation of the conducted syntheses.
14	Reporting bias assessment	To assess the risk of bias, syntheses were performed independently by multiple reviewers.
15	Certainty assessment	To assess certainty in the body of evidence, syntheses were performed independently by multiple reviewers.

**Table 2:** The appearance of data in the official WHO dataset.

*DATE*	*CC*	Country	*WHO_R_*	*C_N_*	*C_C_*	*D_N_*	*D_C_*
…							
14/02/2021	DZ	Algeria	AFRO	210	110,513	3	2935
15/02/2021	DZ	Algeria	AFRO	198	110,711	4	2939
16/02/2021	DZ	Algeria	AFRO	183	110,894	4	2943
17/02/2021	DZ	Algeria	AFRO	175	111,069	2	2945
03/01/2020	AS	American Samoa	WPRO	0	0	0	0
04/01/2020	AS	American Samoa	WPRO	0	0	0	0
05/01/2020	AS	American Samoa	WPRO	0	0	0	0
…							

**Table 3:** An example of the data contained in the JHU dataset.

Province/ State	Country/ Region	Lat	Long	1/22	1/23	…	3/21	3/22	…
	Thailand	15	101	2	3		411	599	
	Japan	36	138	2	1		1007	1086	
	Singapore	1.2833	103.8333	0	1		432	455	
	Nepal	28.1667	84.25	0	0		1	2	
	Malaysia	2.5	112.5	0	0	…	1183	1306	…
British Columbia	Canada	49.2827	−123.121	0	0		424	424	
Victoria	Australia	−37.8136	144.9631	0	0		229	296	
Queensland	Australia	−28.0167	153.4	0	0		221	221	

**Table 4:** An example of data contained in the ECDC dataset.

Date	*C*	*D*	*Country*	*geoID*	*CC*	*POP*	Continent	Cumulative for 14 Days per 100,000
…								
25/07/2020	157	1	China	CN	CHN	$1.43\cdot 10^{9}$	Asia	0.081323
24/07/2020	139	1	China	CN	CHN	$1.43\cdot 10^{9}$	Asia	0.073163
23/07/2020	135	0	China	CN	CHN	$1.43\cdot 10^{9}$	Asia	0.066677
22/07/2020	74	2	China	CN	CHN	$1.43\cdot 10^{9}$	Asia	0.059563
21/07/2020	84	0	China	CN	CHN	$1.43\cdot 10^{9}$	Asia	0.055866
20/07/2020	130	0	China	CN	CHN	$1.43\cdot 10^{9}$	Asia	0.051751
19/07/2020	80	1	China	CN	CHN	$1.43\cdot 10^{9}$	Asia	0.043661
…								

**Table 5:** Result comparison for feed-forward ANN-based algorithms.

Paper	Method	Metric	Result		
[69]	MLP	$R^{2}$	Confirmed	0.94	
			Deceased	0.986	
			Recovered	0.781	
[87]	ANN	MSE	557,422		
		MAE	23.85		
[71]	ANN	Getis-Ord Gi*	*p* < 0.05		
[75]	MLP (ISA-MFN)	$R^{2}$	USA	13,131.18	
			Italy	2757.33	
			Spain	5748.39	
		RMSE	USA	0.92	
			Italy	0.99	
			Spain	0.96	
[76]	ANN	$R^{2}$	0.99		
		MSE	$3.7160\times 10^{-5}$		
[77]	MLP	$R^{2}$	7.75		
		RMSE	10.41		
		MAE	0.99		
[78]	MLP	MSE	West Java	Active	36.29
				Confirmed	29.05
				Recovered	2.19
				Deceased	0.06
			Jakarta	Active	3.21
				Confirmed	19.78
				Recovered	39.24
				Deceased	0.72
[79]	ANN	$R^{2}$	0.76		
[80]	MLP	Accuracy	0.87		
		$F_{1}$	0.92		
		Precision	0.87		
		Recall	0.99		
		AUC	0.63		
[81]	ANN Ensemble	RMSE	Confirmed	1554.03	
			Deceased	162.16	
		MSE	Confirmed	2,415,010.11	
			Deceased	26,297.27	
[82]	ANN	MAE	0.0144		
		MAPE	13.29%		
[85]	MLP	MRE	4.20%		

**Table 6:** Result comparison for RNN-based algorithms.

Paper	Method	Metric	Result		
[88]	RNN	RMSLE	0.5		
[95]	LSTM	MRE	Brazil	3.66%	
			Singapore	2.39%	
			New Zeland	0.74%	
			Taiwan	0.40%	
			Finland	10.7%	
[96]	LSTM	MSE	Saudi Arabia	Confirmed	0.8111
				Deceased	0.0914
			Italy	Confirmed	0.1426
				Deceased	0.09302
			Spain	Confirmed	0.0572
				Deceased	0.01355
		RMSE	Saudi Arabia	Confirmed	0.9006
				Deceased	0.3024
			Italy	Confirmed	0.3777
				Deceased	0.0196
			Spain	Confirmed	0.2296
				Deceased	0.1164
		Mean Error	Saudi Arabia	Confirmed	0.8811
				Deceased	0.1290
			Italy	Confirmed	0.3585
				Deceased	0.2991
			Spain	Confirmed	0.1949
				Deceased	0.0364
[97]	LSTM	RMSE	Saudi Arabia	111.52	
			Sweden	1756.58	
			Argentina	2795.88	
			Indonesia	3691.23	
[98]	LSTM	RMSE	601.2		
[99]	LSTM	RMSE	27.187		
[100]	LSTM	Normalized RMSE	0.01		
[101]	LSTM	RMSE	Confirmed	1103.5	
			Recovered	329	
			Deceased	101.9	
[103]	LSTM	RMSE	947.7027		
[106]	LSTM	RMSE	63.88		
[108]	LSTM	RMSE	34.83		
			45.70		
		Accuracy	93.4		
			92.6		
[109]	LSTM	RMSE	6 neurons	2994.85	
			1 neuron	3331.93	
[110]	LSTM	AUC	0.625		
		$F_{1}$	0.9189		
[111]	LSTM	MAPE	Weekly	8%	
			Daily	3%	

**Table 7:** Result comparison for EC-based algorithms.

Paper	Method	Metrics	Score	
[139]	GP	$R^{2}$	Confirmed	0.9999
			Deaths	0.9997
		RMSE	Confirmed	5.5574
			Deceased	90.1863
[140]	GP	$R^{2}$	Confirmed	0.9999
			Deceased	0.9994
[141]	GA	MAE	45.079011	
[142]	GA	MRE	5%	
[149]	GA	Mean Aboslute Deviation	3385.65	
		MSE	20,050,015.56	
		RMSE	4477.72	
		MAPE	9.68	
[151]	GP	$R^{2}$	Confirmed	0.999
			Recovered	0.998
			Deceased	0.999
[153]	GP	$R^{2}$	Active for entire USA	0.9933
[152]	MAPE	0.114		

## Data Availability

The datasets analyzed in this study are: World Health Organization COVID-19 Dashboard (https://covid19.who.int/, accessed on 14 April 2021), COVID-19 Data Repository by Johns Hopkins University (https://github.com/CSSEGISandData/COVID-19, accessed on 14 April 2021), European Centre for Disease Prevention and Control Daily Number of New Reported COVID-19 Cases and Deaths Worldwide (https://www.ecdc.europa.eu/en/publications-data/download-todays-data-geographic-distribution-covid-19-cases-worldwide, accessed on 14 April 2021), and Worldometer COVID-19 Coronavirus (https://www.worldometers.info/, accessed on 14 April 2021).

## Abbreviations

The following abbreviations are used in this manuscript:

AI	Artificial Intelligence
ANN	Artificial Neural Network
AE	Auto Encoder
ARIMA	Auto-Regressive Integrated Moving Average
BIC	Bayesian Information Criteria
CDC	Center for Disease Control and Prevention
CSSE	Center for System Science and Engineering
CL	Chaotic Learning
CNN	Convolutional Neural Network
CUBIST	Cubist Regression
DE	Differential Evolution
EBK	Empirical Bayesian Kriging
EEMD	Ensemble Empirical Mode Decomposition
EC	Evolutionary Computation
ES	Exponential Smoothing
ELM	Extreme Learning Machine
FF	Feed Forward
FPA	Flower Polination Algorithm
GRU	Gate Recurrent Unit
GLP	Gaussian Learning Process
GWO	Gray Wolf Optimizer
NBC	Gaussian Naïve Bayes
GPR	Gaussian Process Regression
GA	Genetic Algorithm
GP	Genetic Programming
GNN	Graph Neural Network
GMDH	Group Method of Data Handling
IANFIS	Imporved Adaptive Neuro-Fuzzy Inference System
ICA	Imperialist Competitive Algorithm
ISA	Interior Search Algorithm
JHU	John Hopkins University
LASSO	Least Absolute Shrinkage and Selection
LR	Linear Regression
LSTM	Long Short-Term Memory
ML	Machine Learning
MPA	Marine Predators Algorithm
MAE	Mean Absolute Arror
MAPE	Mean Absolute Percentage Error
MRE	Mean Relative Error
MSE	Mean Square Error
MFNN	Multilayer feed-forward Neural Network
MLP	Multilayer Perceptron
NHC	National Health Commission of the People’s Republic of China
NN	Neural Network
NARNN	Nonlinear Autoregression Neural Network
NAR	Non-Linear Autoregressive
PCA	Principal Component Analysis
PSO	Particle Swarm Optimization
$R^{2}$	Coefficient of Determination
RF	Random Forest
RNN	Recurrent Neural Network
RT	Regression Tree
RIDGE	Ridge Regression
RMSE	Root Mean Square Error
RMSRE	Root Mean Square Relative Error
RMSLE	Root Mean Squared Logarithmic Error
SSA	Salp Swarm Algorithm
STAR	Smooth Transition Autoregressive Models
SVM	Support Vector Machine
SVR	Support Vector Regression
SEIR	Suspectible-Exposed-Infectious-Removed
SIR	Suspectible-Infected-Recovered
TAR	Threshold Autoregressive Models
VOA	Virus Optimization Algorithm
VC	Voting Classifier
WBF	Wavelet Based Forecasting
WHO	World Health Organization

## References

[1] Wu D., Wu T., Liu Q., Yang Z. (2020). The SARS-CoV-2 outbreak: What we know. Int. J. Infect. Dis..

[2] Hu B., Guo H., Zhou P., Shi Z.L. (2020). Characteristics of SARS-CoV-2 and COVID-19. Nat. Rev. Microbiol..

[3] Lu H., Stratton C.W., Tang Y.W. (2020). The Wuhan SARS-CoV-2—What’s next for China. J. Med. Virol..

[4] Giesen C., Diez-Izquierdo L., Saa-Requejo C.M., Lopez-Carrillo I., Lopez-Vilela C.A., Seco-Martlinez A., Prieto M.T.R., Malmierca E., Garcia-Fernandez C., Surveillance C.E. (2021). Epidemiological characteristics of the COVID-19 outbreak in a secondary hospital in Spain. Am. J. Infect. Control..

[5] Saez M., Tobias A., Varga D., Barceló M.A. (2020). Effectiveness of the measures to flatten the epidemic curve of COVID-19. The case of Spain. Sci. Total Environ..

[6] Matrajt L., Leung T. (2020). Evaluating the effectiveness of social distancing interventions to delay or flatten the epidemic curve of coronavirus disease. Emerg. Infect. Dis..

[7] Štifanić D., Musulin J., Miočević A., Baressi Šegota S., Šubić R., Car Z. (2020). Impact of covid-19 on forecasting stock prices: An integration of stationary wavelet transform and bidirectional long short-term memory. Complexity.

[8] Dwivedi Y.K., Hughes D.L., Coombs C., Constantiou I., Duan Y., Edwards J.S., Gupta B., Lal B., Misra S., Prashant P. (2020). Impact of COVID-19 pandemic on information management research and practice: Transforming education, work and life. Int. J. Inf. Manag..

[9] Nižetić S. (2020). Impact of coronavirus (COVID-19) pandemic on air transport mobility, energy, and environment: A case study. Int. J. Energy Res..

[10] Tsallis C., Tirnakli U. (2020). Predicting COVID-19 peaks around the world. Front. Phys..

[11] Vespignani A., Tian H., Dye C., Lloyd-Smith J.O., Eggo R.M., Shrestha M., Scarpino S.V., Gutierrez B., Kraemer M.U., Wu J. (2020). Modelling covid-19. Nat. Rev. Phys..

[12] Ziff A.L., Ziff R.M. (2020). Fractal kinetics of COVID-19 pandemic. MedRxiv.

[13] Seemann T., Lane C.R., Sherry N.L., Duchene S., da Silva A.G., Caly L., Sait M., Ballard S.A., Horan K., Schultz M.B. (2020). Tracking the COVID-19 pandemic in Australia using genomics. Nat. Commun..

[14] Thurner S., Klimek P., Hanel R. (2020). A network-based explanation of why most COVID-19 infection curves are linear. Proc. Natl. Acad. Sci. USA.

[15] Holmdahl I., Buckee C. (2020). Wrong but useful—What covid-19 epidemiologic models can and cannot tell us. N. Engl. J. Med..

[16] Fahrmeir L., Kneib T., Lang S., Marx B. (2007). Regression.

[17] Higgins J., Green S. (2020). Meta-regression. Cochrane Handb. Syst. Rev. Interv. Version.

[18] Tu J.V. (1996). Advantages and disadvantages of using artificial neural networks versus logistic regression for predicting medical outcomes. J. Clin. Epidemiol..

[19] Zhou Y., Ma Z., Brauer F. (2004). A discrete epidemic model for SARS transmission and control in China. Math. Comput. Model..

[20] Bhatt D., Vyas D., Kumhar M., Patel A. (2019). Swine Flu Predication Using Machine Learning. Information and Communication Technology for Intelligent Systems.

[21] Ganasegeran K., Abdulrahman S.A. (2020). Artificial intelligence applications in tracking health behaviors during disease epidemics. Human Behaviour Analysis Using Intelligent Systems.

[22] Gulyaeva M., Huettmann F., Shestopalov A., Okamatsu M., Matsuno K., Chu D.H., Sakoda Y., Glushchenko A., Milton E., Bortz E. (2020). Data mining and model-predicting a global disease reservoir for low-pathogenic Avian Influenza (A) in the wider pacific rim using big data sets. Sci. Rep..

[23] Martcheva M. (2014). Avian flu: Modeling and implications for control. J. Biol. Syst..

[24] Nan Y., Gao Y. (2018). A machine learning method to monitor China’s AIDS epidemics with data from Baidu trends. PLoS ONE.

[25] Bisaso K.R., Anguzu G.T., Karungi S.A., Kiragga A., Castelnuovo B. (2017). A survey of machine learning applications in HIV clinical research and care. Comput. Biol. Med..

[26] Learning F.U.M. (2018). Performance Analysis of Time Series Forecasting Using Machine Learning Algorithms for Prediction of Ebola Casualties. Proceedings of the Applications of Computing and Communication Technologies: First International Conference, ICACCT 2018.

[27] Bhattacharyya D., Kumari N.M.J., Joshua E.S.N., Rao N.T. (2020). Advanced Empirical Studies on Group Governance of the Novel Corona Virus, MERS, SARS and EBOLA: A Systematic Study. Int. J. Curr. Res. Rev..

[28] Vaishya R., Javaid M., Khan I.H., Haleem A. (2020). Artificial Intelligence (AI) applications for COVID-19 pandemic. Diabetes Metab. Syndr. Clin. Res. Rev..

[29] Naudé W. (2020). Artificial Intelligence Against COVID-19: An Early Review.

[30] Tayarani-N M.H. (2020). Applications of artificial intelligence in battling against Covid-19: A literature review. Chaos Solitons Fractals.

[31] Agbehadji I.E., Awuzie B.O., Ngowi A.B., Millham R.C. (2020). Review of big data analytics, artificial intelligence and nature-inspired computing models towards accurate detection of COVID-19 pandemic cases and contact tracing. Int. J. Environ. Res. Public Health.

[32] Adly A.S., Adly A.S., Adly M.S. (2020). Approaches based on artificial intelligence and the internet of intelligent things to prevent the spread of COVID-19: Scoping review. J. Med. Internet Res..

[33] Ahmad A., Garhwal S., Ray S.K., Kumar G., Malebary S.J., Barukab O.M. (2020). The number of confirmed cases of covid-19 by using machine learning: Methods and challenges. Arch. Comput. Methods Eng..

[34] Page M.J., McKenzie J.E., Bossuyt P.M., Boutron I., Hoffmann T.C., Mulrow C.D., Shamseer L., Tetzlaff J.M., Moher D. (2021). Updating guidance for reporting systematic reviews: Development of the PRISMA 2020 statement. J. Clin. Epidemiol..

[35] Friedman J., Hastie T., Tibshirani R. (2001). The Elements of Statistical Learning.

[36] Bishop C.M. (2006). Pattern Recognition and Machine Learning.

[37] Goodfellow I., Bengio Y., Courville A., Bengio Y. (2016). Deep Learning.

[38] Centre for Disease Control USA. https://www.cdc.gov/.

[39] Robert Koch Institute. https://www.rki.de.

[40] Ministero Della Salute. http://www.salute.gov.it/portale/nuovocoronavirus/.

[41] Instituto de Salud Carlos III. https://www.isciii.es/Paginas/Inicio.aspx.

[42] National Health Commission of the People’s Republic of China. http://www.nhc.gov.cn.

[43] Brazil Health Ministry. http://www.brazil.gov.br/government/ministers/health.

[44] World Health Organization Homepage. https://www.who.int/.

[45] WHO Coronavirus Disease (Covid-19) Dashboard. https://covid19.who.int/.

[46] John Hopkins University Homepage. https://www.jhu.edu/.

[47] Dong E., Du H., Gardner L. (2020). An Interactive Web-Based Dashboard to Track COVID-19 in Real Time. Lancet Infect. Dis..

[48] COVID-19 Dashboard by the Center for Systems Science and Engineering (CSSE) at Johns Hopkins University (JHU). https://www.arcgis.com/apps/opsdashboard/index.html/bda7594740fd40299423467b48e9ecf6.

[49] COVID-19 Data Repository by the Center for Systems Science and Engineering (CSSE) at Johns Hopkins University. https://github.com/CSSEGISandData/COVID-19.

[50] European Centre for Disease Prevention and Control Homepage. https://www.ecdc.europa.eu/en.

[51] Download Historical Data (to 14 December 2020) on the Daily Number of New Reported COVID-19 Cases and Deaths Worldwide. https://www.ecdc.europa.eu/en/publications-data/download-todays-data-geographic-distribution-covid-19-cases-worldwide.

[52] Worldometer COVID-19 Coronavirus Pandemic. https://www.worldometers.info/coronavirus/.

[53] Muhammad L., Islam M.M., Usman S.S., Ayon S.I. (2020). Predictive data mining models for novel coronavirus (COVID-19) infected patients’ recovery. SN Comput. Sci..

[54] Plohl N., Musil B. (2021). Modeling compliance with COVID-19 prevention guidelines: The critical role of trust in science. Psychol. Health Med..

[55] Signorelli C., Scognamiglio T., Odone A. (2020). COVID-19 in Italy: Impact of containment measures and prevalence estimates of infection in the general population. Health.

[56] Toulis P. (2021). Estimation of COVID-19 prevalence from serology tests: A partial identification approach. J. Econom..

[57] Medema G., Heijnen L., Elsinga G., Italiaander R., Brouwer A. (2020). Presence of SARS-Coronavirus-2 RNA in sewage and correlation with reported COVID-19 prevalence in the early stage of the epidemic in the Netherlands. Environ. Sci. Technol. Lett..

[58] Brown T.S., Walensky R.P. (2020). Serosurveillance and the COVID-19 Epidemic in the US: Undetected, Uncertain, and Out of Control. JAMA.

[59] COVID-19 Serology Surveillance. https://www.cdc.gov/coronavirus/2019-ncov/covid-data/serology.

[60] Coronavirus (COVID-19) Testing. https://ourworldindata.org/coronavirus-testing.

[61] Muñoz-Fernández G.A., Seoane J.M., Seoane-Sepúlveda J.B. (2021). A SIR-type model describing the successive waves of COVID-19. Chaos Solitons Fractals.

[62] Sedaghat A., Oloomi S.A.A., Malayer M.A., Band S.S., Mosavi A., Nadai L. (2020). Modeling and Sensitivity Analysis of Coronavirus Disease (COVID-19) Outbreak Prediction. https://www.medrxiv.org/content/10.1101/2020.11.18.20234419v1.

[63] Ivorra B., Ferrández M.R., Vela-Pérez M., Ramos A. (2020). Mathematical modeling of the spread of the coronavirus disease 2019 (COVID-19) taking into account the undetected infections. The case of China. Commun. Nonlinear Sci. Numer. Simul..

[64] Ramos A., Ferrández M., Vela-Pérez M., Kubik A., Ivorra B. (2021). A simple but complex enough *θ*-SIR type model to be used with COVID-19 real data. Application to the case of Italy. Phys. D Nonlinear Phenom..

[65] Badr H.S., Du H., Marshall M., Dong E., Squire M.M., Gardner L.M. (2020). Association between mobility patterns and COVID-19 transmission in the USA: A mathematical modelling study. Lancet Infect. Dis..

[66] Malavika B., Marimuthu S., Joy M., Nadaraj A., Asirvatham E.S., Jeyaseelan L. (2021). Forecasting COVID-19 epidemic in India and high incidence states using SIR and logistic growth models. Clin. Epidemiol. Glob. Health.

[67] Singh P., Gupta A. (2021). Generalized SIR (GSIR) epidemic model: An improved framework for the predictive monitoring of COVID-19 pandemic. ISA Trans..

[68] Moein S., Nickaeen N., Roointan A., Borhani N., Heidary Z., Javanmard S.H., Ghaisari J., Gheisari Y. (2021). Inefficiency of SIR models in forecasting COVID-19 epidemic: A case study of Isfahan. Sci. Rep..

[69] Car Z., Baressi Šegota S., Anđelić N., Lorencin I., Mrzljak V. (2020). Modeling the spread of COVID-19 infection using a multilayer perceptron. Comput. Math. Methods Med..

[70] Sujath R., Chatterjee J.M., Hassanien A.E. (2020). A machine learning forecasting model for COVID-19 pandemic in India. Stoch. Environ. Res. Risk Assess..

[71] Chakraborty T., Ghosh I. (2020). Real-time forecasts and risk assessment of novel coronavirus (COVID-19) cases: A data-driven analysis. Chaos Solitons Fractals.

[72] Chen L.P. (2020). Analysis and Prediction of Covid-19 Data in Taiwan. https://papers.ssrn.com/sol3/papers.cfm?abstract_id=3611761.

[73] Mollalo A., Rivera K.M., Vahedi B. (2020). Artificial neural network modeling of novel coronavirus (COVID-19) incidence rates across the continental United States. Int. J. Environ. Res. Public Health.

[74] Kumar P., Kalita H., Patairiya S., Sharma Y.D., Nanda C., Rani M., Rahmani J., Bhagavathula A.S. (2020). Forecasting the dynamics of COVID-19 pandemic in top 15 countries in April 2020: ARIMA model with machine learning approach. medRxiv.

[75] Rizk-Allah R.M., Hassanien A.E. (2020). COVID-19 forecasting based on an improved interior search algorithm and multi-layer feed forward neural network. arXiv.

[76] Hasan N. (2020). A methodological approach for predicting COVID-19 epidemic using EEMD-ANN hybrid model. Internet Things.

[77] Saba A.I., Elsheikh A.H. (2020). Forecasting the prevalence of COVID-19 outbreak in Egypt using nonlinear autoregressive artificial neural networks. Process. Saf. Environ. Prot..

[78] Pontoh R.S., Toharudin T., Zahroh S., Supartini E. (2020). Effectiveness of the public health measures to prevent the spread of covid-19. Commun. Math. Biol. Neurosci..

[79] Vaid S., Cakan C., Bhandari M. (2020). Using machine learning to estimate unobserved COVID-19 infections in North America. J. Bone Jt. Surgery. Am..

[80] Alakus T.B., Turkoglu I. (2020). Comparison of deep learning approaches to predict COVID-19 infection. Chaos Solitons Fractals.

[81] Melin P., Monica J.C., Sanchez D., Castillo O. (2020). Multiple ensemble neural network models with fuzzy response aggregation for predicting COVID-19 time series: The case of Mexico. Healthcare.

[82] Baltas G., Prieto Rodríguez F.A., Frantzi M., García Alonso C., Rodríguez Cortés P. (2020). Monte Carlo Deep Neural Network Model for Spread and Peak Prediction of COVID-19.

[83] Farooq J., Bazaz M.A. (2020). A novel adaptive deep learning model of Covid-19 with focus on mortality reduction strategies. Chaos Solitons Fractals.

[84] Pereira I.G., Guerin J.M., Silva Júnior A.G., Garcia G.S., Piscitelli P., Miani A., Distante C., Gonçalves L.M.G. (2020). Forecasting Covid-19 dynamics in Brazil: A data driven approach. Int. J. Environ. Res. Public Health.

[85] Ndiaye B.M., Tendeng L., Seck D. (2020). Analysis of the COVID-19 pandemic by SIR model and machine learning technics for forecasting. arXiv.

[86] Pinter G., Felde I., Mosavi A., Ghamisi P., Gloaguen R. (2020). COVID-19 pandemic prediction for Hungary; A hybrid machine learning approach. Mathematics.

[87] Moftakhar L., Mozhgan S., Safe M.S. (2020). Exponentially increasing trend of infected patients with COVID-19 in Iran: A comparison of neural network and ARIMA forecasting models. Iran. J. Public Health.

[88] Tomar A., Gupta N. (2020). Prediction for the spread of COVID-19 in India and effectiveness of preventive measures. Sci. Total. Environ..

[89] Shoaib M., Raja M.A.Z., Sabir M.T., Bukhari A.H., Alrabaiah H., Shah Z., Kumam P., Islam S. (2021). A stochastic numerical analysis based on hybrid NAR-RBFs networks nonlinear SITR model for novel COVID-19 dynamics. Comput. Methods Programs Biomed..

[90] Kolozsvari L.R., Berczes T., Hajdu A., Gesztelyi R., TIba A., Varga I., Szollosi G.J., Harsanyi S., Garboczy S., Zsuga J. (2020). Predicting the epidemic curve of the coronavirus (SARS-CoV-2) disease (COVID-19) using artificial intelligence. medRxiv.

[91] Tamang S., Singh P., Datta B. (2020). Forecasting of covid-19 cases based on prediction using artificial neural network curve fitting technique. Glob. J. Environ. Sci. Manag..

[92] Direkoglu C., Sah M. (2020). Worldwide and regional forecasting of coronavirus (covid-19) spread using a deep learning model. medRxiv.

[93] Chimmula V.K.R., Zhang L. (2020). Time series forecasting of COVID-19 transmission in Canada using LSTM networks. Chaos Solitons Fractals.

[94] Arora P., Kumar H., Panigrahi B.K. (2020). Prediction and analysis of COVID-19 positive cases using deep learning models: A descriptive case study of India. Chaos Solitons Fractals.

[95] Chatterjee A., Gerdes M.W., Martinez S.G. (2020). Statistical explorations and univariate timeseries analysis on covid-19 datasets to understand the trend of disease spreading and death. Sensors.

[96] Hartono P. (2020). Similarity maps and pairwise predictions for transmission dynamics of covid-19 with neural networks. Inform. Med. Unlocked.

[97] Aldhyani T.H., Alrasheed M., Alzahrani M.Y., Ahmed H. (2020). Deep learning and Holt-trend algorithms for predicting COVID-19 pandemic. medRxiv.

[98] Yudistira N. (2020). COVID-19 growth prediction using multivariate long short term memory. arXiv.

[99] Vadyala S.R., Betgeri S.N., Sherer E.A., Amritphale A. (2020). Prediction of the number of covid-19 confirmed cases based on k-means-lstm. arXiv.

[100] Ayyoubzadeh S.M., Ayyoubzadeh S.M., Zahedi H., Ahmadi M., Kalhori S.R.N. (2020). Predicting COVID-19 incidence through analysis of google trends data in iran: Data mining and deep learning pilot study. JMIR Public Health Surveill..

[101] Pal R., Sekh A.A., Kar S., Prasad D.K. (2020). Neural network based country wise risk prediction of COVID-19. Appl. Sci..

[102] Zhao Z., Nehil-Puleo K., Zhao Y. (2020). How well can we forecast the COVID-19 pandemic with curve fitting and recurrent neural networks?. medRxiv.

[103] Kırbaş İ., Sözen A., Tuncer A.D., Kazancıoğlu F.Ş. (2020). Comparative analysis and forecasting of COVID-19 cases in various European countries with ARIMA, NARNN and LSTM approaches. Chaos Solitons Fractals.

[104] Dutta H. (2020). Neural Network Model for Prediction of Covid-19 Confirmed Cases and Fatalities.

[105] The COVID Tracking Project. https://covidtracking.com/data/api.

[106] Tian Y., Luthra I., Zhang X. (2020). Forecasting COVID-19 cases using Machine Learning models. medRxiv.

[107] Yan B., Tang X., Liu B., Wang J., Zhou Y., Zheng G., Zou Q., Lu Y., Tu W. (2020). An improved method of COVID-19 case fitting and prediction based on LSTM. arXiv.

[108] Pirouz B., Shaffiee Haghshenas S., Shaffiee Haghshenas S., Piro P. (2020). Investigating a serious challenge in the sustainable development process: Analysis of confirmed cases of COVID-19 (new type of coronavirus) through a binary classification using artificial intelligence and regression analysis. Sustainability.

[109] Javid A.M., Liang X., Venkitaraman A., Chatterjee S. (2020). Predictive analysis of covid-19 time-series data from johns hopkins university. arXiv.

[110] Huang C.J., Chen Y.H., Ma Y., Kuo P.H. (2020). Multiple-input deep convolutional neural network model for covid-19 forecasting in china. medRxiv.

[111] Shaffiee Haghshenas S., Pirouz B., Shaffiee Haghshenas S., Pirouz B., Piro P., Na K.S., Cho S.E., Geem Z.W. (2020). Prioritizing and analyzing the role of climate and urban parameters in the confirmed cases of COVID-19 based on artificial intelligence applications. Int. J. Environ. Res. Public Health.

[112] Ribeiro M.H.D.M., da Silva R.G., Mariani V.C., dos Santos Coelho L. (2020). Short-term forecasting COVID-19 cumulative confirmed cases: Perspectives for Brazil. Chaos Solitons Fractals.

[113] Vaid S., McAdie A., Kremer R., Khanduja V., Bhandari M. (2020). Risk of a second wave of Covid-19 infections: Using artificial intelligence to investigate stringency of physical distancing policies in North America. Int. Orthop..

[114] Tuli S., Tuli S., Tuli R., Gill S.S. (2020). Predicting the growth and trend of COVID-19 pandemic using machine learning and cloud computing. Internet Things.

[115] Melin P., Monica J.C., Sanchez D., Castillo O. (2020). Analysis of spatial spread relationships of coronavirus (COVID-19) pandemic in the world using self organizing maps. Chaos Solitons Fractals.

[116] Kapoor A., Ben X., Liu L., Perozzi B., Barnes M., Blais M., O’Banion S. (2020). Examining covid-19 forecasting using spatio-temporal graph neural networks. arXiv.

[117] Rustam F., Reshi A.A., Mehmood A., Ullah S., On B.W., Aslam W., Choi G.S. (2020). COVID-19 future forecasting using supervised machine learning models. IEEE Access.

[118] Poonia N., Azad S. (2020). Short-term forecasts of COVID-19 spread across Indian states until 1 May 2020. arXiv.

[119] Khan H.R., Hossain A. (2020). Countries are clustered but number of tests is not vital to predict global covid-19 confirmed cases: A machine learning approach. medRxiv.

[120] Cabras S. (2020). A bayesian-deep learning model for estimating covid-19 evolution in spain. arXiv.

[121] Ndiaye B.M., Tendeng L., Seck D. (2020). Comparative prediction of confirmed cases with COVID-19 pandemic by machine learning, deterministic and stochastic SIR models. arXiv.

[122] Yahia N.B., Kandara M.D., Saoud N.B.B. (2020). Deep Ensemble Learning Method to Forecast COVID-19 Outbreak.

[123] Wang P., Zheng X., Li J., Zhu B. (2020). Prediction of epidemic trends in COVID-19 with logistic model and machine learning technics. Chaos Solitons Fractals.

[124] Onovo A., Atobatele A., Kalaiwo A., Obanubi C., James E., Gado P., Odezugo G., Ogundehin D., Magaji D., Russell M. (2020). Using Supervised Machine Learning and Empirical Bayesian Kriging to Reveal Correlates and Patterns of Covid-19 Disease Outbreak in Sub-Saharan Africa: Exploratory Data Analysis. https://papers.ssrn.com/sol3/Papers.cfm?abstract_id=3580721.

[125] Chaurasia V., Pal S. (2020). Covid-19 Pandemic: Application of Machine Learning Time Series Analysis for Prediction of Human Future. https://papers.ssrn.com/sol3/papers.cfm?abstract_id=3652378.

[126] Stochiţoiu R.D., Rebedea T., Popescu I., Leordeanu M. (2020). A self-supervised neural-analytic method to predict the evolution of covid-19 in romania. arXiv.

[127] Al-Qaness M.A., Ewees A.A., Fan H., Abd El Aziz M. (2020). Optimization method for forecasting confirmed cases of COVID-19 in China. J. Clin. Med..

[128] Malki Z., Atlam E.S., Hassanien A.E., Dagnew G., Elhosseini M.A., Gad I. (2020). Association between weather data and COVID-19 pandemic predicting mortality rate: Machine learning approaches. Chaos Solitons Fractals.

[129] Gupta A., Gharehgozli A. (2020). Developing a Machine Learning Framework to Determine the Spread of COVID-19. https://papers.ssrn.com/sol3/papers.cfm?abstract_id=3635211.

[130] Velásquez R.M.A., Lara J.V.M. (2020). Forecast and evaluation of COVID-19 spreading in USA with reduced-space Gaussian process regression. Chaos Solitons Fractals.

[131] Uhlig S., Nichani K., Uhlig C., Simon K. (2020). Modeling projections for COVID-19 pandemic by combining epidemiological, statistical, and neural network approaches. medRxiv.

[132] Pasayat A.K., Pati S.N., Maharana A. (2020). Predicting the COVID-19 positive cases in India with concern to Lockdown by using Mathematical and Machine Learning based Models. medRxiv.

[133] The Humanitarian Data Exchange. https://data.humdata.org/.

[134] Al-Qaness M.A., Ewees A.A., Fan H., Abualigah L., Abd Elaziz M. (2020). Marine predators algorithm for forecasting confirmed cases of COVID-19 in Italy, USA, Iran and Korea. Int. J. Environ. Res. Public Health.

[135] Abhari R.S., Marini M., Chokani N. (2020). Covid-19 epidemic in switzerland: Growth prediction and containment strategy using artificial intelligence and big data. medRxiv.

[136] Erraissi A., Banane M. (2020). Machine Learning model to predict the number of cases contaminated by COVID-19. Int. J. Comput. Digit. Syst..

[137] Carrillo-Larco R.M., Castillo-Cara M. (2020). Using country-level variables to classify countries according to the number of confirmed COVID-19 cases: An unsupervised machine learning approach. Wellcome Open Res..

[138] Niazkar M., Niazkar H.R. (2020). Covid-19 outbreak: Application of multi-gene genetic programming to country-based prediction models. Electron. J. Gen. Med..

[139] Salgotra R., Gandomi M., Gandomi A.H. (2020). Time series analysis and forecast of the COVID-19 pandemic in India using genetic programming. Chaos Solitons Fractals.

[140] Salgotra R., Gandomi M., Gandomi A.H. (2020). Evolutionary modelling of the COVID-19 pandemic in fifteen most affected countries. Chaos Solitons Fractals.

[141] Rayungsari M., Aufin M., Imamah N. (2020). Parameters estimation of generalized richards model for covid-19 cases in indonesia using genetic algorithm. Jambura J. Biomath. (JJBM).

[142] Rouabah M.T., Tounsi A., Belaloui N.E. (2020). Early dynamics of COVID-19 in Algeria: A model-based study. arXiv.

[143] Yousefpour A., Jahanshahi H., Bekiros S. (2020). Optimal policies for control of the novel coronavirus disease (COVID-19) outbreak. Chaos Solitons Fractals.

[144] Behnood A., Golafshani E.M., Hosseini S.M. (2020). Determinants of the infection rate of the COVID-19 in the US using ANFIS and virus optimization algorithm (VOA). Chaos Solitons Fractals.

[145] Howard D. (2020). Genetic Programming visitation scheduling solution can deliver a less austere COVID-19 pandemic population lockdown. arXiv.

[146] Ghosh S., Bhattacharya S. (2020). A data-driven understanding of COVID-19 dynamics using sequential genetic algorithm based probabilistic cellular automata. Appl. Soft Comput..

[147] Hosseini E., Ghafoor K.Z., Sadiq A.S., Guizani M., Emrouznejad A. (2020). Covid-19 optimizer algorithm, modeling and controlling of coronavirus distribution process. IEEE J. Biomed. Health Inform..

[148] Higazy M., Alyami M.A. (2020). New Caputo-Fabrizio fractional order SEIASqEqHR model for COVID-19 epidemic transmission with genetic algorithm based control strategy. Alex. Eng. J..

[149] Elmousalami H.H., Hassanien A.E. (2020). Day level forecasting for Coronavirus Disease (COVID-19) spread: Analysis, modeling and recommendations. arXiv.

[150] Kwuimy C., Nazari F., Jiao X., Rohani P., Nataraj C. (2020). Nonlinear dynamic analysis of an epidemiological model for COVID-19 including public behavior and government action. Nonlinear Dyn..

[151] Anđelić N., Baressi Šegota S., Lorencin I., Mrzljak V., Car Z. (2021). Estimation of COVID-19 epidemic curves using genetic programming algorithm. Health Inform. J..

[152] Ardabili S., Mosavi A., Band S.S., Varkonyi-Koczy A.R. (2020). Coronavirus Disease (COVID-19) Global Prediction Using Hybrid Artificial Intelligence Method of ANN Trained with Grey Wolf Optimizer. medRxiv.

[153] Anđelić N., Baressi Šegota S., Lorencin I., Jurilj Z., Šušteršič T., Blagojević A., Protić A., Ćabov T., Filipović N., Car Z. (2021). Estimation of COVID-19 Epidemiology Curve of the United States Using Genetic Programming Algorithm. Int. J. Environ. Res. Public Health.

[154] Pedregosa F., Varoquaux G., Gramfort A., Michel V., Thirion B., Grisel O., Blondel M., Prettenhofer P., Weiss R., Dubourg V. (2011). Scikit-learn: Machine learning in Python. J. Mach. Learn. Res..

[155] Wang W., Wang S., Gao J., Zhang M., Chen G., Ng T.K., Ooi B.C. (2018). Rafiki: Machine learning as an analytics service system. arXiv.

[156] Ketkar N. (2017). Introduction to keras. Deep Learning with Python.

[157] Hochreiter S., Schmidhuber J. (1997). Long short-term memory. Neural Comput..

[158] Song X., Liu Y., Xue L., Wang J., Zhang J., Wang J., Jiang L., Cheng Z. (2020). Time-series well performance prediction based on Long Short-Term Memory (LSTM) neural network model. J. Pet. Sci. Eng..

[159] Nagelkerke N.J. (1991). A note on a general definition of the coefficient of determination. Biometrika.

[160] Saunders L.J., Russell R.A., Crabb D.P. (2012). The coefficient of determination: What determines a useful R2 statistic?. Investig. Ophthalmol. Vis. Sci..

